# Niche differentiation within bacterial key-taxa in stratified surface waters of the Southern Pacific Gyre

**DOI:** 10.1093/ismejo/wrae155

**Published:** 2024-08-03

**Authors:** Monike Oggerin, Tomeu Viver, Jan Brüwer, Daniela Voß, Marina García-Llorca, Oliver Zielinski, Luis H Orellana, Bernhard M Fuchs

**Affiliations:** Department of Molecular Ecology, Max Planck Institute for Marine Microbiology, Bremen D-28359, Germany; Department of Molecular Ecology, Max Planck Institute for Marine Microbiology, Bremen D-28359, Germany; Department of Molecular Ecology, Max Planck Institute for Marine Microbiology, Bremen D-28359, Germany; Institute of Chemistry and Biology of the Marine Environment, University of Oldenburg, Wilhelmshafen, Germany; Department of Molecular Ecology, Max Planck Institute for Marine Microbiology, Bremen D-28359, Germany; Institute of Chemistry and Biology of the Marine Environment, University of Oldenburg, Wilhelmshafen, Germany; Leibniz Institute for Baltic Sea Research Warnemünde, D-18119 Rostock, Germany; Department of Molecular Ecology, Max Planck Institute for Marine Microbiology, Bremen D-28359, Germany; Department of Molecular Ecology, Max Planck Institute for Marine Microbiology, Bremen D-28359, Germany

**Keywords:** Aegean169, metagenomics, metatranscriptomics, stratification

## Abstract

One of the most hostile marine habitats on Earth is the surface of the South Pacific Gyre (SPG), characterized by high solar radiation, extreme nutrient depletion, and low productivity. During the SO-245 “UltraPac” cruise through the center of the ultra-oligotrophic SPG, the marine alphaproteobacterial group AEGEAN169 was detected by fluorescence *in situ* hybridization at relative abundances up to 6% of the total microbial community in the uppermost water layer, with two distinct populations (*Candidatus* Nemonibacter and *Ca.* Indicimonas). The high frequency of dividing cells combined with high transcript levels suggests that both clades may be highly metabolically active. Comparative metagenomic and metatranscriptomic analyses of AEGEAN169 revealed that they encoded subtle but distinct metabolic adaptions to this extreme environment in comparison to their competitors SAR11, SAR86, SAR116, and *Prochlorococcus*. Both AEGEAN169 clades had the highest percentage of transporters per predicted proteins (9.5% and 10.6%, respectively). In particular, the high expression of ABC transporters in combination with proteorhodopsins and the catabolic pathways detected suggest a potential scavenging lifestyle for both AEGEAN169 clades. Although both AEGEAN169 clades may share the genomic potential to utilize phosphonates as a phosphorus source, they differ in their metabolic pathways for carbon and nitrogen. *Ca.* Nemonibacter potentially use glycine-betaine, whereas *Ca.* Indicimonas may catabolize urea, creatine, and fucose. In conclusion, the different potential metabolic strategies of both clades suggest that both are well adapted to thrive resource-limited conditions and compete well with other dominant microbial clades in the uppermost layers of SPG surface waters.

## Introduction

One of Earth’s most extreme marine habitats is the South Pacific Gyre (SPG), covering 10% of the world’s oceans. The SPG is the farthest away from any land mass; thus, it is virtually cut off from the supply of many vital nutrients. These conditions result in a permanent hyper-oligotrophic state at its center with extremely low nutrient concentrations [1, 2], extremely low chlorophyll *a* concentrations of ~0.02 mg m^−3^ [3, 4], and consequently low primary productivity [5, 6]. Due to the low productivity, microbial cell counts are among the lowest reported, with values as low as ~4 × 10^5^ cells ml^−1^ in the center of the gyre [7–11]. For these reasons, the SPG is often referred to as the largest oceanic desert on Earth [12–14]. It features the clearest waters on the planet, with high ultraviolet (UV) light transmission to the deepest depths, and is the oceanic waters equivalent of the purest freshwater habitats [15].

To date, only a few scientific research cruises have been devoted to the composition and metabolism of microbial communities in the hyper-oligotrophic SPG. In 2004, the Biosope cruise provided the first insights into the abundance and activity of the main microbial groups in the center of the SPG [8, 12, 16]. At the turn of 2015–2016 during the Ultrapac cruise, data on DOC accumulation, microbial community composition, and primary production were collected in the surface waters of the central gyre [2, 5, 9]. UV and visible light showed very high irradiance rate in water layers at the center of the SPG, with the highest penetration depths at the central gyre (Fig. S1). This may have contributed to the highly stratified microbial communities [9] and the relatively high rate of DOC accumulation detected in surface water layers of the central SPG [2]. Five microbial groups dominated the surface water layer. Besides the bacterial clades SAR11, SAR86, SAR116, and *Prochlorococcus*, all of which are known to be well adapted to oligotrophic environments and have streamlined, small genomes [17–21], a less known clade called AEGEAN169 reached high relative abundances ranging 3%–6% in 20 m water depth [9]. The AEGEAN169 clade was first described in 2005 in the northern Aegean Sea at 200 m depth [22] and has since been found in a variety of marine locations such as the San Pedro Channel (North Pacific) [23–25], Mediterranean Sea [26], Red Sea [27, 28], Atlantic Ocean [29–31], Xiamen Sea [32], Adriatic Sea [33], and SPG [9, 11]. The preferential distribution and abundance of AEGEAN169 in mainly oligo- to hyper-oligotrophic marine habitats indicates that this group appears to be perfectly adapted and has metabolisms that make it competitive with other microbial groups.

In this study, we investigated how the most abundant microbial clades cope with the low nutrient levels, high radiation intensities, and strong competition for substrates in one of Earth’s most extreme pelagic marine environments. We selected samples from the three most central SPG stations and analyzed the microbial communities at the surface and above and below the deep chlorophyll maximum using metagenomic and metatranscriptomic approaches. We focused our analyses on the less known AEGEAN169 clade and its strategies to compete with the other well-described and abundant clades SAR11, SAR116, SAR86, and *Prochlorococcus.* The genomic repertoire of AEGEAN169 has been partially explored [29], but little is known about its metabolic expression profiles in its natural marine habitat. Our results suggest a clear niche partitioning between two AEGEAN169 clades inhabiting the surface waters of the central SPG, and we propose two new candidate genera, *Candidatus* Nemonibacter and *Ca.* Indicimonas, with the following candidate species: *Ca.* Nemonibacter pelagicus, *Ca.* Indicimonas nautili, *Ca.* Indicimonas poseoidonii, and *Ca.* Indicimonas neptunia.

## Material and Methods

### Sample collection

Seawater samples were collected at stations SO245-04 (23.5° S, 100° W), SO245-06 (23.48° S, 110.03° W), and SO245-08 (27.73° WS, 117.62° W) during the UltraPac expedition (SO-245) aboard the R/V Sonne from Antofagasta, Chile (17 December 2015) to Wellington, New Zealand (28 January 2016) across the SPG (see [9] for details). For this work, we selected three depths for all stations, corresponding at 20 m below surface, above the deep chlorophyll maximum (aDCM, 140 m for stations 4 and 8, 170 m for station 6), and below the DCM (bDCM, 170 m at station 4, 200 m at stations 6 and 8). Water was taken with *in situ* large volume pumps (Large Water Transfer System, McLane) and filtered onto 142 mm polyethersulfone membranes composed of two membranes of 0.8 μm pore size put on top of each other until clogging for 20 m depth and bDCM and 0.2 μm pore size polycarbonate filter for aDCM, respectively (Table S1). Samples were stored at −80°C until processing.

### DNA, RNA extraction, and sequencing

DNA and RNA were extracted sequentially from the same samples using the AllPrep Bacterial DNA/RNA/Protein Kit (Qiagen, Germany). Metagenomes were sequenced on the Hiseq 2500 platform (Illumina, San Diego, USA) with paired-end sequencing [34]. For station 6 and 8 from 20 m depth, additional samples were sequenced on the PacBio sequel II platform (Menlo Park, USA). Metatranscriptomes, were sequenced on the Hiseq 3000 platform with pair-end sequencing at the Max Planck Genome Center in Cologne (Germany).

### Metagenome assembly and binning

Quality-filtered reads from each metagenome were individually assembled using IDBA-UD v1.1.3 [35] with kmer lengths from 51 to 121. Subsampling (10% and 25%) of reads from metagenomes [36] corresponding to 20 m depth, aDCM, or bDCM of all stations was performed using BBtools v38.79 [37]. Resulting subsamples were assembled as described above, retaining contigs longer than 1 kpb for binning. Maxbin2 v2.2.4 [38], Metabat2 v2.12 [39], and Concoct v1.0.0 [40] within the Metawrap suit v1.0 [41] were used for binning. Reads from depth-related metagenomes were mapped back to assemblies to increase sequencing depth and binning accuracy. Bin refinement was done with the Metawrap suit v1.0. The obtained bins were quality-filtered (% completion − 5*(% contamination) > 50). Bins with a completeness higher than 70% were selected for further analyses. Further details and statistics of the SPG metagenomes and metatranscriptomes are provided in the supplementary file Table S2. Metagenome-assembled genomes (MAGs) from this study and published references were dereplicated using dRep v2.4 [42] (ANI cutoff 95%) and taxonomically classified using GTDB-tk v1.7.0 [43] with the reference database version r202 [44].

### Relative abundance

Reconstructed MAGs and external references were analyzed by mapping Illumina short reads using Bowtie2 v2.4.2 [45] with default settings. Reads with 99% identity were filtered using the *Sam.filter.rb* script from the enveomics collection [46]. Genomecov from the Bedtools package [47] was used to determine sequencing depth. TAD80 values were calculated using the *BedGraph.tad.rb* script from the enveomics collection and then compared to genome equivalents using MicrobeCensus [48, 49] to determine the fraction of each genome in the microbial community.

### Metagenome-assembled genomes and references used in this study

Despite using various assembly and binning methods, no AEGEAN169 bins were recovered from either Illumina or the long-read PacBio sequel II metagenomic sequences, even after subsampling. To address this, we used previously published AEGEAN169 single amplified genomes (SAGs) [50, 51] identified from GTDB r202 to recruit reads from our metagenomic samples. High coverage rates were achieved using the 80% central truncated average of the sequencing depth (TAD80) criteria [49]. This strategy was extended to other targeted clades to identify potential genomes present at the central gyre. For phylogenetic analysis, the genomes listed in Table S3 were used.

### Gene prediction and annotation

Protein prediction was performed using Prodigal v2.6.3 [52]. Functional annotation was done with EggNOG-mapper v2.1.0 [53] based on eggNOG orthology data [54]. Sequence searches were performed using DIAMOND v2.0.2 [55] with default settings. Annotations were confirmed using selected TIGRFAM and PFAM HMM models (Table S4) using HMMER3.3 [56]. Results required of at least 70% of the length of the model length with an evalue <1e-20. CAZyme annotation was performed using the hmmscan function (evalue <1e-18) and dbCAN v9 database with the *hmmscan-parser.sh* script [57, 58]. The accuracy of the annotations in the SPG was verified by manual examination of the BAM files derived from the alignment of the SPG metagenomes to reference genomes.

### Metatranscriptomic analyses

Messenger-RNA (mRNA) reads were extracted from quality-filtered metatranscriptomes using SortMeRNA v2.1 [59] with default settings. Reads were then mapped to selected genomes and those >95% identity were pooled for each bacterial clade after removing duplicates using SeqKit toolkit v0.15.0 [60]. Proteins were predicted from the short reads using FragGeneScan [61] with default settings. TIGRFAM and PFAM HMM models were used (evalue <1e-5, quotient of the hmm aligned model sequence and predicted protein fragment length > 0.8 and unique predicted protein sequences selected) to generate raw counts for each clade at each station and depth. Biological scaling normalization (BSN) was utilized as normalization method to ensure comparability among samples [62]. Housekeeping genes involved in primary metabolic activities (*atpA, gyrA, ftsZ, glnA, grpE, recA, rpoB, rpoD, prfA, ychF, tig, tsf, coaE, infC, if-2, frr*) [63, 64] were calculated and used as reference metabolic state to determine the fold change of target genes.

## Results and Discussion

### Surface South Pacific Gyre community is dominated by five major clades and is strongly stratified

**Figure 1:**
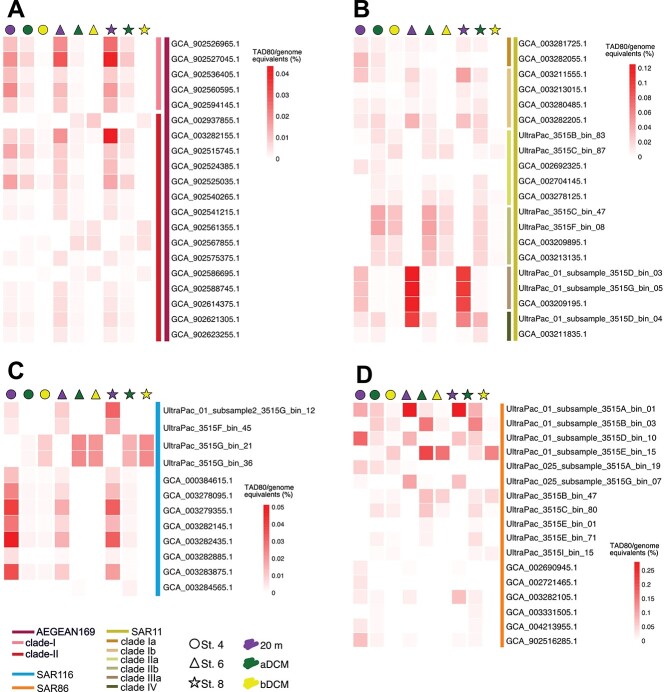
Relative abundance of part of the genomes detected in the SPG at stations (St.) 4 (circle), 6 (triangle), and 8 (star). (A) AEGEAN169. (B) SAR11. (C) SAR116. (D) SAR86. The pronounced partitioning of the different microorganisms within the water column is clearly visible. TAD80—truncated average sequencing depth using the middle 80% of the sequence base positions.

The binning process of 11 individual SPG metagenomes, including 9 Illumina and 2 PacBio Sequel II sequenced metagenomes from the center of the SPG, resulted in 1130 bins (Table S2). After two rounds of refinement and dereplication at 95% ANI, the number of MAGs was reduced to 311 belonging to 18 different phyla (Table S2, Fig. S2A), of which 212 had >70% completeness and <5% contamination. Depth distribution was pronounced in the non-metric multidimensional scaling (NMDS) metagenomic analysis (Fig. S2B) and for AEGEAN169, SAR11, SAR116, and SAR86 genomes distribution (Fig. 1, Fig. S3). This strong stratification is consistent with previous results from the SPG [9] and may be influenced by the intense UV and VIS irradiance detected at the central gyre, potentially affecting and displacing the DCM (Fig. S1). This stratification reveals that genomes of SAR11 clades Ia, Ib, and IIIa are mainly identified within the first 20 m water depth and above the DCM. However, SAR11 clades IIa and IIb were absent at 20 m and present only in water layers above and below the DCM (see Table S5, Fig. 1B). Similar distribution patterns were observed for SAR86 (Fig. 1D) and SAR116 (Fig. 1C). Likewise, several AEGEAN169 genomes were detected exclusively at 20 m depth across all stations (Fig. 1A, Table S5), whereas another AEGEAN169 subpopulation was identified mainly above and below the DCM. These results suggest a pronounced depth-dependent distribution of the microbial community within the central SPG, not at the genus level but with distinct species-level preferences for specific depth layers. This phenomenon raises the question of whether this depth-dependent pattern is unique to the SPG or represents a more general characteristic of oligotrophic ocean gyres. Future studies are warranted to investigate this phenomenon in more detail.

### Two distinct AEGEAN169 species are abundant in the surface South Pacific Gyre

**Figure 2:**
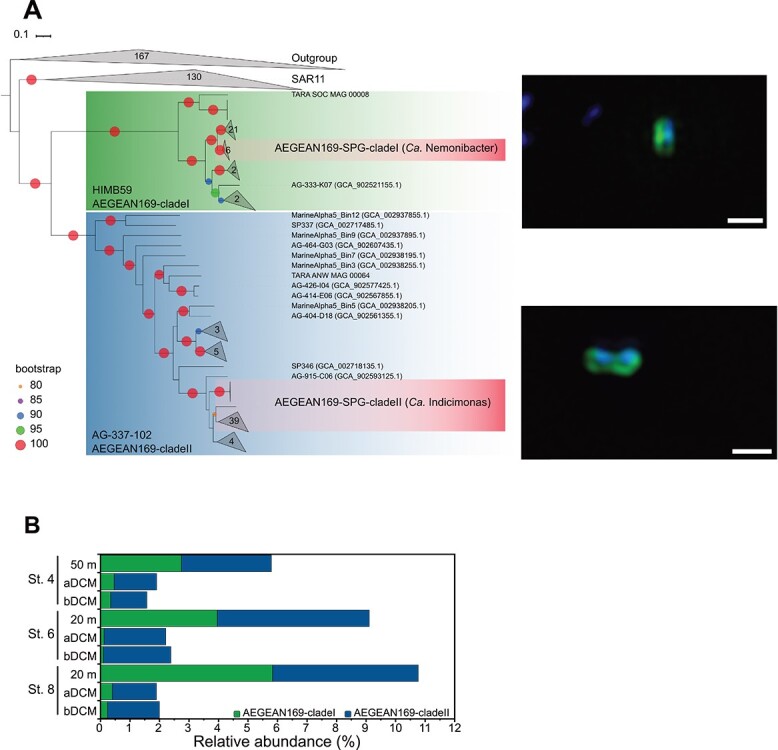
(A) RAxML phylogenomic reconstruction of genomes belonging to AEGEAN169 clades, with SAR11, using SAR116, SAR86, *Prochlorococcus* and selected Alphaproteobacteria type strains of marine origin as out-group, calculated with 1000 bootstrap replicates. Collapsed AEGEAN169 clades represent all clades whose average branch length distance to their leaves is below 0.2. Insets: Super-resolution structured illumination microscopy micrographs of AEGEAN169 clade I and clade II cells, respectively. White bars bottom right in each micrograph represent 0.5 μm. (B) Relative abundance plot of the AEGEAN169 at the central SPG stations (St.) 4, 6, and 8 using the specific AEGEAN169 probes. Abbreviations: aDCM—above-deep chlorophyll *a* maximum; bDCM—below-deep chlorophyll *a* maximum.

To further disentangle the AEGEAN169 populations from the SPG, we performed a phylogenetic analysis that highlighted the presence of two distinct clades *Ca.* Nemonibacter and *Ca.* Indicimonas (Fig. 2A, Fig. S3). The two clades were also observed from the 16S rRNA gene sequence analysis (Fig. S4). To further characterize single units or species for each clade, we used the mapping of short reads to representative genomes. A high intrapopulation sequence diversity was evident for the clades (Fig. S5A and B). The number of reads per similarity value peaks between 97% and 94%, followed by a noticeable decrease toward 90%. Thus, the average nucleotide identity of mapping reads (ANIr) to GCA_902527045.1 (*Ca.* Nemonibacter) and GCA_003282155.1 (*Ca.* Indicimonas) genomes of ~90% constituted a precise threshold for distinguishing sequence-discrete populations. This method identified four species in the uppermost water layers (first 20 m) of the central SPG.

Pairwise average amino acid identity (AAI) comparisons between *Ca.* Nemonibacter and *Ca.* Indicimonas members revealed values consistently exceeding 80% within each clade, with values <50% between clades, suggesting that the clades detected in the SPG may correspond to two genera (Fig. S5E). In contrast, pairwise AAI values calculated between the AEGEAN169 and SAR11 genomes had an average AAI of ~41.6%, strongly suggesting that both groups may indeed belong to different orders [65, 66]. Consequently, it can be inferred that the AEGEAN169 marine clade is not affiliated with the *Pelagibacteraceae* family. This result aligns with previous research, which consistently indicated that HIMB59 (AEGEAN169) does not belong to the SAR11 clade and, therefore, cannot be considered a member of the *Pelagibacteraceae* family [67–70] (Fig. S5F).

Relative cell abundances for both AEGEAN169 clades were the highest at 20–50 m depth across all stations ranging from 2.8% to 5.8% for AEGEAN169-clade I and ~5% for AEGEAN169-clade II. Nonetheless, abundances decreased with depth to 0.2% for clade I and ~1.2%–2% for clade II below DCM (Fig. 2B). An increased prevalence of dividing cells (~5%) was observed for both clades within the upper 20 m at all stations, contrasting with a decreasing frequency of dividing cells at greater depths. This decrease correlated with depth, reaching 1% for AEGEAN169-clade I and 2.5% for AEGEAN169-clade II in the deeper layers. The highest number of transcripts belonging to *Ca.* Nemonibacter and *Ca.* Indicimonas were found in 20 m depth at all stations (Fig. S6). Thus, the combination of metagenomics, FISH abundances, and metatranscriptomic analysis suggests that both *Ca.* Nemonibacter and *Ca.* Indicimonas are abundant, metabolically active, and preferentially inhabit the first 20 m water column of the central SPG.

### *Ca.* Nemonibacter and *Ca.* Indigobacter may assimilate CO_2_ supported by photo pigments

**Figure 3:**
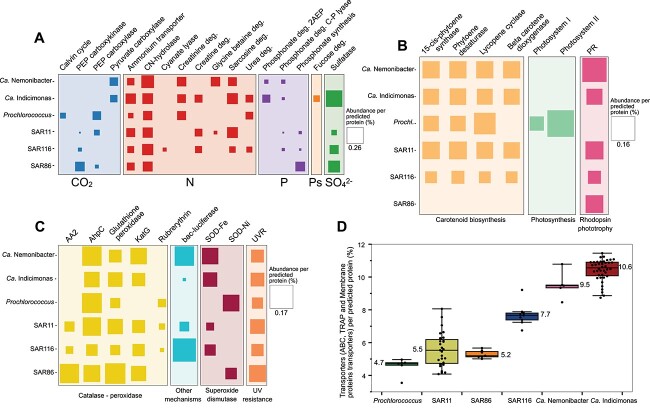
HMM model searches showing the number of distinct features per predicted protein. (A) shows an exploration of C/N/P pathways across different clades of the SPG. (B) illustrates various protein models associated with phototrophic metabolism. (C) highlights the protein models specifically related to oxidative stress defense in SPG clades. (D) represents the percentage of total transporter systems in relation to the total number of predicted proteins that were detected within each SPG clade. Abbreviations: deg. (degradation); PEP (phosphoenolpyruvate); 2AEP (2-aminoethylphosphonate); PR (proteorhodopsin); AA2 (auxiliary-activity-2 enzyme); AhpC (alkyl-hydroperoxide-reductase C); KatG (bifunctional catalase-peroxidase); SOD-Fe (superoxide dismutase containing iron); SOD-Ni (superoxide dismutase containing nickel); UVR (UV repair and resistance); ABC (ATP synthase-binding cassette transporter), TRAP (tripartite ATP-independent periplasmic transporter).

Proteorhodopsins (PRs) in *Ca.* Nemonibacter and *Ca.* Indicimonas, SAR11, SAR116, and SAR86 SPG genomes suggest their dependence on light to support their metabolism in SPG surface waters (Fig. 3B, Tables S7–S11). Predicted protein sequence alignments suggested a blue-absorbing PR for all examined clades (Fig. S7). Phylogenetic analysis grouped *Ca.* Indicimonas PRs into a single cluster and *Ca.* Nemonibacter PRs as a sister cluster to the SAR116 PRs. A second *Ca.* Nemonibacter PR suggested as a green-absorbing PR grouped in a distant cluster [71] (Figs S7 and S8). These results agree with recent findings reported for the entire AEGEAN169 clade [29]. As light-dependent proton pumps, PRs may have the potential to meet the energy demands required to sustain the basal metabolism at the central SPG using the proton motive force through the anaplerotic CO_2_ assimilation to utilize the tricarboxylic acid (TCA) cycle for biosynthesis by replenishing the cycle intermediates [72–74]. Three out of the four known potential anaplerotic CO_2_ assimilation pathways were detected in the investigated clades (Fig. S9). The pyruvate carboxylase pathway was found only in *Ca.* Nemonibacter and *Ca.* Indicimonas genomes, whereas the phosphoenolpyruvate (PEP) carboxylase and PEP carboxykinase pathways were identified in the competing SPG clades SAR11, SAR116, SAR86, and *Prochlorococcus* (Fig. 3A, Tables S7–S11).

**Figure 4:**
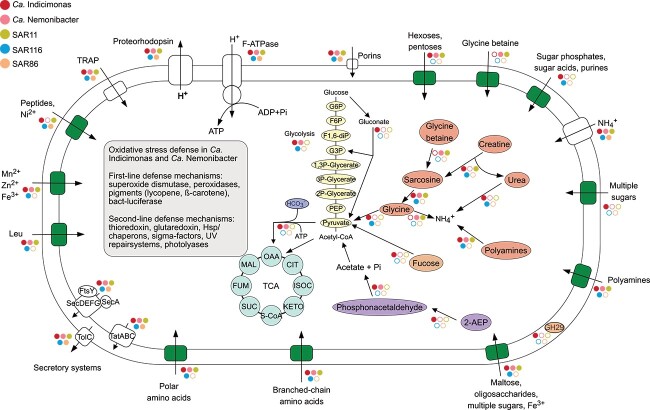
Representation of potential pathways and transport systems operating within the distinct clades inhabiting the uppermost 20 m of the central SPG. This outline provides insight into the potential metabolic processes and transport mechanisms these microorganisms utilize in the central SPG. Solid circles indicate the presence of the feature, white circles denote its absence. Abbreviations: TRAP (tripartite ATP-independent periplasmic transporter); G6P (glucose-6-phosphate) F6P (fructose 6-phosphate); F1,6-dIP (fructose 1,6-bi-phosphate); G3P (glyceraldehyde 3-phosphate); PEP (phosphoenolpyruvate); TCA (tricarboxylic acid cycle); OAA (oxaloacetate); CIT (citrate); ISOC (isocitrate); KETO (α-ketoglutarate); S-CoA (succinyl-CoA); SUC (succinate); FUM (fumarate); MAL (malate).

Genomic potential of the core metabolic functions in *Ca.* Nemonibacter and *Ca.* Indicimonas suggests a heterotrophic lifestyle (Table S6). Both SPG clades may be capable of glycolysis, and parts of the semiphosphorylative Entner–Doudorof pathway were detected in some *Ca.* Indicimonas members, but not in *Ca.* Nemonibacter. The TCA cycle and the nonoxidative pentose pathway were detected in both clades (Fig. 4). Manual inspection and curation of AEGEAN169 genomes revealed that the SAGs from different oligotrophic regions share the same metabolic potential with respect to the pathways under study, regardless of the geographic origin.

### *Ca.* Nemonibacter and *Ca.* Indicimonas may have the capability to utilize alternative N and P sources

**Figure 5:**
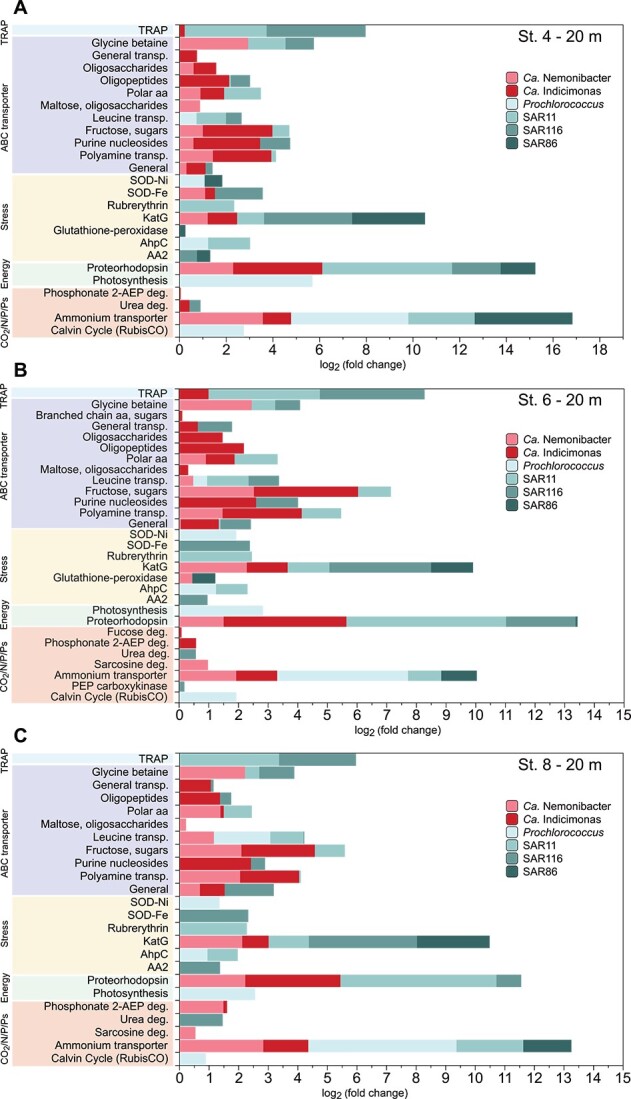
Protein models with the highest expression levels calculated relative to the reference metabolic state across different metabolic pathways. The values shown are positive log2 (fold-change) values, and refer to the first 20 m of the SPG stations and clades studied. (A) Station 4. (B) Station 6. (C) Station 8. These bar plots provide a comprehensive view of the transcriptional activity associated with different metabolic processes at different SPG stations and within different clades, shedding light on their metabolic responses in this unique marine environment. Abbreviations: TRAP (tripartite ATP-independent periplasmic transporter); polar AA (polar amino acids); SOD-Ni (superoxide dismutase containing nickel); SOD-Fe (superoxide dismutase containing iron); KatG (bifunctional catalase-peroxidase); AhpC (alkyl hydroperoxide reductase C); AA2 (auxiliary-activity-2 enzyme); deg. (degradation); 2-AEP deg. (2-aminoethylphosphonate degradation); RubisCO (ribulose-1,5-bisphosphate carboxylase/oxygenase); PEP carboxykinase (phosphoenolpyruvate carboxykinase).

Extremely low concentrations of dissolved inorganic N and P characterize the SPG. None of the clades examined carry the genetic potential for N fixation or assimilatory NO_3_^−^/NO_2_^−^ reduction. However, ammonium transporters were detected in all investigated clades, particularly *Ca.* Indicimonas genomes carried up to two transporters per genome (Fig. 3A, Tables S7–S11). Our hypothesis suggests that to overcome the scarcity of N and P, the AEGEAN169 marine group may have developed strategies to acquire these elements from the abundant marine reservoirs of dissolved organic nitrogen (DON) and dissolved organic phosphorus (DOP). Urea, consistently found in different marine habitats, emerges as a crucial nitrogen source among DON compounds [75, 76]. Genes responsible for urea utilization were found exclusively in *Ca.* Indicimonas (present in 80% of members), all *Prochlorococcus*, and SPG SAR116 (60%). Sarcosine, another potential nitrogen source, can be metabolized by bacteria to glycine to be used for biosynthesis or cleaved into ammonium and CO_2_ [77, 78]. Sarcosine is a central product in several metabolic pathways and may be produced during creatine degradation in *Ca.* Indicimonas or glycine betaine (GBT) catabolism in *Ca.* Nemonibacter [77, 78] (Fig. 5, Table S7). Creatine is a compatible solute of phytoplankton and metazoan origin [79]. We detected the sarcosine oxidase genes in both *Ca.* Nemonibacter and *Ca.* Indicimonas, which encode for a key enzyme of the sarcosine utilization (Fig. 3A, Table S7). Metatranscriptomic analysis reveals relatively high levels of transcripts related to the sarcosine oxidation in *Ca.* Nemonibacter (Fig. 4B and C), suggesting that its degradation may contribute to glycine production, which could be used for biosynthesis or as a source of ammonium and pyruvate [80]. GBT catabolism, a rare phenomenon in prokaryotes [78], previously reported only in some SAR11, HIMB59, and *Rhodovulum sulfidophilum* (*Rhodobacterales*) [78], was identified in all *Ca.* Nemonibacter members and a few SPG SAR11 members belonging to clades Ia, Ib, and IV (Fig. 3A, Tables S7 and S8). Indeed, one of the most highly expressed ABC transporters in *Ca.* Nemonibacter was the specific ABC transporter for GBT (OpuAC) (Fig. 4A–C). Consequently, GBT may serve as a source of N and pyruvate, providing *Ca.* Nemonibacter with a competitive advantage in the ultra-oligotrophic SPG.

Polyamines are also important components of the DON reservoir and serve as an N source for marine bacteria by integrating their carbon skeleton into the TCA cycle [81]. Polyamine transporters were identified in *Ca.* Nemonibacter, *Ca.* Indicimonas, SPG-SAR11, and two SPG-SAR116 (Fig. S10, Tables S7–S9).

Phosphonates play a crucial role as a P source for marine microorganisms, especially in nutrient-poor regions such as the SPG, where they can constitute up to 25% of the DOP in the SPG water column [82]. In fact, ~10% of marine bacteria have the potential to degrade phosphonates through different pathways and to use them as a P source [83, 84]. A prominent pathway for phosphonate degradation is the C-P lyase pathway, which is widespread among marine bacteria due to its lack of substrate specificity [85]. This pathway, consisting of the genes *phnK*, *phnL*, *phnH*, *phnG,* and *phnJ*, was only identified in a limited number of SPG members, including one *Ca.* Nemonibacter, five *Ca.* Indicimonas, one SAR11 (clade Ia), and two SPG SAR116 members (Fig. 3A, Tables S7–S9). The expression of enzymes involved in this pathway remained consistently below the metabolic reference level for all clades and stations. In contrast, the 2-aminoethylphosphonate (2-AEP) hydrolytic pathway, specific for the most abundant phosphonate compound in marine environments, 2-AEP [86], leading to the production of acetate and phosphate was prevalent in *Ca.* Indicimonas (over 85% of the members). In addition, 30% of *Ca.* Nemonibacter members encoded a partial 2-AEP degradation pathway, containing genes for the last two steps of the pathway (*phnY* and *phnA*) (Fig. 3A, Table S7). Transcripts related to different steps of the 2-AEP degradation pathway were detected above the metabolic reference level in *Ca.* Indicimonas at all stations and in *Ca.* Nemonibacter at station 8 (Fig. 4A–C). Consistent with previous studies [84, 87], these results suggest that phosphonates may serve as source of P and C for both *Ca.* Indicimonas and *Ca.* Nemonibacter.

Our hypothesis suggest that both *Ca.* Nemonibacter and *Ca.* Indicimonas may be specialized in degrading a range of phyto- and zooplankton-derived compounds as N and P sources, most likely from decaying biomass. *Ca.* Indicimonas has the metabolic potential to degrade creatine and sarcosine, with key enzymes detected in over 85% of tested genomes. In contrast, *Ca.* Nemonibacter possesses enzymes for the degradation of GBT and sarcosine; GBT may act as an alternative source of sarcosine rather than creatine (Fig. 4). Both clades seem to be able to use phosphonates and are therefore well equipped to utilize various N and P sources at the inhospitable SPG.

### *Ca.* Indicimonas may be potential fucose degraders

To investigate whether the SPG AEGEAN169 might play a role in the degradation of polysaccharides in the SPG, we examined the genetic potential annotated as carbohydrate-active enzymes. A glycosyl hydrolase of family 29 (GH29) annotated as exo-fucosidase was detected only in 20% of the *Ca.* Indicimonas representatives (Fig. 3A, Table S7). A single copy of the GH29 gene per SAG was detected, which is part of the nonphosphorylating fucose degradation pathway [88, 89] (Fig. S11A and B). The fucose utilization operon containing the GH29 gene had a conserved gene arrangement in *Ca.* Indicimonas (Fig. S12). This fucose utilization operon was absent in the remainder of the AEGEAN169 marine group, and it was not present in any of the other SPG clades.

Fucose-containing sulfated polysaccharides (FCSPs) are often decorated with sulfate moieties to prevent easy digestion [90]. In order to serve as substrates, sulfatases are needed to cleave the sulfate esters to gain access to the polysaccharide backbone containing the fucose [91]. In *Ca.* Indicimonas representatives, an average of five sulfatases per genome was found, ranging from two to eight, indicating that they were not enriched in sulfatases. CAZymes necessary for the full degradation of FCSP were apparently absent in contrast to other specialized FCSP degraders such as members of the Verrucomicrobiota [49, 92]. Our analyses indicate that although *Ca.* Indicimonas may use fucose, it seems unlikely that it utilizes polymeric FCSP directly. Transcripts related to this pathway were detected at all stations, although most of the times, the expression of this pathway was below the reference state with values of log_2_ fold-change ranging from ~−2 to −3 as an average. However, one of the steps of the pathway (2-keto-3-deoxy-L-fuconate dehydrogenase) could be detected slightly above that limit with a value of 0.1 in station 6 (Fig. 5B). We believe that *Ca.* Indicimonas is likely to rely on the activity of other organisms to degrade FCSP, but this hypothesis and how *Ca.* Indicimonas ultimately gets access to fucose need to be explored in further experiments. Nevertheless, fucose seems to be a potential extra carbon source, which may be only accessible to the *Ca.* Indicimonas among all competing SPG clades.

### Highest number of transporters in *Ca.* Nemonibacter and *Ca.* Indicimonas

In nutrient-poor oligotrophic environments, a common strategy among oligotrophic microbial clades is to rely on a variety of transport systems to efficiently meet their nutritional requirements [93, 94]. A prime example is HIMB59 (AEGEAN169 clade I, formerly included in the SAR11 clade as clade V), which is known to have the highest ratio of transport systems per protein-coding gene compared to all other bacteria [95]. In the SPG, both AEGEAN169 clades, *Ca.* Nemonibacter and *Ca.* Indicimonas, had an exceptional abundance of transporters compared to all other competing clades in both absolute and relative terms as transporters per megabase pair (Mbp) exceeded the values for SAR11 by a factor of two (Fig. 3D, Tables S7–S11). Of particular importance were the ABC transporters, which accounted for a substantial fraction, reaching up to 5.8% and 7.4% in *Ca.* Nemonibacter and *Ca.* Indicimonas, respectively, compared to 3% in SAR11 (Fig. S13). Transcript analysis highlighted the potential different transporter utilization strategies of the major clades in the central SPG. For instance, *Ca.* Indicimonas seemed to have the highest diversity of different highly expressed ABC transporters compared to other SPG clades, including *Ca.* Nemonibacter (Fig. 5). These transporters covered a spectrum of substrates, including oligosaccharides and sugars (SBP_bac_1, SBP_bac_8, Peripla_BP_4, BDP_transp_2), purine nucleosides (Bmp), oligopeptides (SBP_bac_5), branched-chain and polar amino acids (BPD_transp_2, SBP_bac_3), and polyamines (Polyamine-transp) (Fig. S10). Another notable group of highly expressed potential ABC transporters in *Ca.* Nemonibacter, *Ca.* Indicimonas, and SAR11 were those specific for polyamines (see above). Although the concentration of various polyamines was not measured in the water column during the SPG cruise, the literature suggests their abundance in marine habitats, particularly putrescine and spermidine, which are often associated with phytoplankton and bacterial production [96, 97]. Further analysis using the Transporter Classification Database (TCDB) for the membrane transport proteins [98] revealed the presence of porins, classified as the major intrinsic protein (MIP) family, involved in the transport of cations and other compounds in *Ca.* Nemonibacter and *Ca.* Indicimonas (Table S7). *Ca.* Nemonibacter and *Ca.* Indicimonas had similar abundance values, ~3.1%–3.8% of the total predicted proteins in each genome, exceeding SAR11 (~2.2%) (Fig. S13).

Most of the detected genes expressed above the reference state were related to transport systems. SAR11 and SAR116 seemed to exhibit different transporter utilization strategies in the SPG, with the most highly expressed transporter system being tripartite Adenosine triphosphate (ATP)-independent periplasmic (TRAP) transporters in both clades (Fig. 5). Unlike ABC transporters, TRAP transporters operate independently of ATP, using ion-electrochemical gradients to drive compounds into the cell in a symporter mechanism [99]. These transporters operate with a wide range of substrates, typically those containing at least one carboxylate group, such as pyruvate, which is the primary substrate for SAR11 [100].

### Surface South Pacific Gyre bacteria are well protected against ultraviolet irradiation damage by a series of quenching and repair mechanisms

UV radiation penetrates deep into the central SPG, potentially damaging biomolecules but is also generating reactive oxygen species (ROS). Therefore, surface-dwelling microorganisms in the SPG, including both *Ca.* Nemonibacter and *Ca.* Indicimonas as well as other clades, are likely to possess effective mechanisms to counteract oxidative stress. We investigated these defense systems in the resident genomes and their expression and identified several known defense mechanisms.

Superoxide dismutases (SODs) are key metalloenzymes responsible for the conversion of superoxide radical (O_2 **˙**_^−^) to H_2_O_2_. Of the three different classes examined (CuZn, Fe/Mn, or Ni containing SOD), most of the SPG members had one of them (Fig. 3C, Tables S7–S11). Peroxidases and catalases, which are involved in the detoxification of H_2_O_2_, were also investigated. Although the genetic potential related to catalase activity was absent in the studied clades, the bifunctional catalase-peroxidase (KatG) was found in all of them except in *Prochlorococcus* (Fig. 3C, Tables S7–S11). In addition, several enzymes with peroxidase activity were identified, including glutathione peroxidase and alkyl hydroperoxide reductase C (AhpC), both identified in all SPG clades. The Auxiliary-Activity-2 enzyme (AA2, Cazyme nomenclature) with peroxidase activity was not present in *Ca.* Nemonibacter*, Ca.* Indicimonas, or *Prochlorococcus*. An additional enzyme with strong peroxidase activity, rubrerythrin [101], was found only in *Prochlorococcus* and SAR11 (4/28 of the members). In contrast, luciferase, reported as protectant against oxidative stress damage [102–104], was detected in all *Ca.* Nemonibacter members, but only in two *Ca.* Indicimonas, a few SAR11 (7/28), and all SAR116 (Fig. 3C, Tables S7–S9). These findings suggest that all the surface inhabitants of the SPG may use a variety of defense mechanisms to combat UV-induced oxidative stress and survive the highly irradiated SPG.

## Conclusions

Our data suggest that members of AEGEAN169 may be scavenging, photoheterotrophic bacteria well adapted to compete for scarce nutrients in the center of the world’s most oligotrophic gyre. Our comprehensive analyses of the metabolic potential, coupled with metatranscriptomic data, may set *Ca.* Nemonibacter and *Ca.* Indicimonas clearly apart from their competitor clades, defining clear niches based on their specific substrate utilization strategies. *Ca.* Nemonibacter and *Ca.* Indicimonas seem to have the highest number of transporters per total predicted proteins of the bacterial communities described here. Their genomic repertoire is consistent with a lifestyle that utilizes necromass from dead organisms, presumably phyto- and zooplankton, as indicated by the potential to use GBT, creatine, and small osmolytes. The intense solar radiation, especially in the upper 20 m of the water column, also causes increased microbial mortality in the SPG. Previous cruises have shown that the number of microbial cells at the surface is lower than at 30–50 m [9], indicating a potentially higher mortality there. Consequently, the full range of cellular repair mechanisms is required to mitigate the radiation damage caused by the high UV light intensities at the surface of the SPG. At the same time, both *Ca.* Nemonibacter and *Ca.* Indicimonas seem to be able to complement their metabolism with proteorhodopsin proton pumps tuned to two different spectral ranges. Combined with PR-mediated ATP generation, this may help to boost anabolism by utilizing some anaplerotic CO_2_ fixation pathways found in both AEGEAN169 SPG clades. Taken together, all the features found for AEGEAN169-SPG make them highly adapted to cohabit with the other four well-described, codominant clades SAR11, *Prochlorococcus*, SAR86, and SAR116.

## Supplementary Material

suppl_wrae155

## Data Availability

Metagenome, metatranscriptome, and MAGG sequence data are available from the European Nucleotide Archive (accession PRJEB71397).
