# Supplementary material for: Niche differentiation within bacterial key-taxa in stratified surface waters of the Southern Pacific Gyre
Source: ISME J. 2024 Aug 3;18(1):wrae155. doi: 10.1093/ismejo/wrae155 (PMC11366302; doi:10.1093/ismejo/wrae155)
Supplement: suppl_wrae155 [file suppl_wrae155.zip › Supplementary Information - clean.docx]

**Supplementary Information to Niche differentiation within bacterial key-taxa in stratified surface waters of the Southern Pacific Gyre**

**Supplementary Materials and Methods**

**Underwater light field**

A HyperPro II profiling system (Sea-Bird Scientific, former Satlantic, Bellevue, USA) was used to collect hyperspectral underwater light field data in free-falling mode in the visible range (VIS). The system consisted of one hyperspectral irradiance (upward looking) and one hyperspectral radiance sensor (downward looking). A second hyperspectral downward irradiance sensor was mounted on the research vessel for matching above-water irradiance reference measurements. A second profiling system with single wavelengths (MicroPro®, Sea-Bird Scientific, former Satlantic, Bellevue, USA) was used to investigate the shortwave underwater light field with the same configuration of radiometer setup and reference unit onboard. For both instruments, profiles were conducted at each station depending on sea and weather conditions, with deployments at approx. 50 m away from the ship to avoid ship shadow. All sensors were pre-calibrated by the manufacturer and validated prior to the cruise with a reference lamp. Data were recorded with the Sea-Bird Scientific’s SatView® software v2.9.5_7, pre-processed using theSea-Bird Scientific’s ProSoft Processing® software v7.7.19_2 and binned in 1 m depth intervals. A dark correction was made automatically based on shutter measurements by the instrument. Post-processing for the visible range was made in accordance with [1, 2]. The photosynthetic active radiation (PAR) was integrated from 400 to 700 nm, considering the onboard reference measurements.

**Metagenome processing**

Trimming and processing of Illumina raw reads was performed as previously published [3]. The average coverage of the different metagenomic sets from the SPG was calculated with Nonpareil v3.3.1 [4]. Non-metric multidimensional analysis (NMDS) and ANOSIM test were performed on quality filtered reads from each metagenomic sample using Mash v2.0 [5].

**Read recruitment plots and AAI calculation**

Recruitment plots were generated using the RecruitPlotEasy tool [6]. The average amino acid identity (AAI) and the fraction of genes shared between pairs of genomes was determined using BLASTp v2.2.31[7] in the aai.rb script from the enveomics collection with minimum alignment length and minimum identity of 50%.

**Additional genome annotation**

Additional annotation tools were used such as Pfam v34.0 [8] (pfam_scan.pl script using default settings), Interproscan [9], Bakta [10], and Prokka [11]. KEGG profiles were obtained with KofamKOALA [12] and BlastKOALA [13] for AEGEAN169 representatives.

**Phylogenetic reconstruction.**

A phylogenomic reconstruction using 120 aligned and concatenated marker genes according to GTDB-tk v1.7.0 and reference database version r202 was done for the genomes belonging to AEGEAN169 (101), SAR11 (130), SAR86 (56), SAR116 (39), and *Prochlorococcus* (17) clades, supplemented with 55 marine Alphaproteobacteria type strains. The tree was calculated using RAxML v8.2.12 [14] with automatic selection of substitution model and 1000 boostrap replicates (-m PROTGAMMAAUTO -p 1234 -x 1234 -#1000). External MAG and SAG published references were downloaded from NCBI database and previous studies [15] (See Table S3).

***In situ* Hybridization on environmental samples.**

CAtalyzed Reported Deposition- *In situ* Hybridization (CARD-FISH) was performed as described in [16] with specific oligonucleotide probes for both AEGEAN169 clades (AEGEAN169-395 and AEGEAN169-744) [17]. While both probes were previously used in a mix, in this study we applied them separately, since they are specific for *Ca*. Nemonibacter and *Ca*. Indicimonas, respectively. Samples were examined under an automated epifluorescence microscope (Zeiss Axio-plan2 imaging, Carl Zeiss, Oberkochen, Germany) equipped with a monochrome camera (AxioCam MRm, Carl Zeiss Microimaging GmbH, Göttingen, Germany). Cell counts were calculated using the digital image analysis software ACMEtool3 [18]. Micrographs of AEGEAN169 cells were taken under Superresolution Structured Illumination Microscopy on a Zeiss LSM 780 microscope (Carl Zeiss, Oberkochen, Germany). Additionally, the frequency of dividing cells, as a proxy for cell division rates, were calculated using the MicrobeJ plugin v5.13I [19] for imageJ/Fiji v2.1.0 [20]. Dividing cells were identified by two local DAPI maxima, compared to single DAPI maxima as described in [21].

**Supplementary Results and Discussion**

*Description of Ca. Nemonibacter gen. nov*

Nemonibacter (Latin description of the name Captain Nemo from character from *Twenty Thousand Leagues Under the Sea* written by Jules Verne. Also, in reference to point Nemo located in the center of the SPG, the most remote place on the planet.)

Members of the genus *Ca.* Nemonibacter are mesophilic, photoheterotrophic marine bacteria, with a scavenging way of life, with the capability of using glycine betaine and polyamines. Performing glycolysis and using the pyruvate carboxylase for the anaplerotic CO2 fixation. Based on ANI values (90%), 1 species belong to this genus. Genomes belonging to this genus are: GCA_902526965.1; GCA_902527045.1; GCA_902536405.1; GCA_902560595.1; GCA_902594145.1. The GC content for *Ca*. Nemonibacter is between 30% and 31%. Cells are rod shaped with an average cell size of 0.5 ± 0.1 µm (n=23) (Fig. 1 A inset). This genus was detected in the central SPG in densities of 1.3x10^4^ to 2.3x10^4^ cell ml^-1^.

*Description of Ca. Nemonibacter spp.*

*Candidatus* Nemonibacter pelagicus (latin description related to open oceanic waters)

This species was detected in open seawater samples from the central ultra-oligotrophic SPG, mainly, in the top 20 m, in December 2015 and January 2016. The estimated GC content is between 30% and 31%. Genomes belonging to this species are: GCA_902526965.1; GCA_902527045.1; GCA_902536405.1; GCA_902560595.1; GCA_902594145.1; GCA_002690585.1.

*Description of Ca. Indicimonas gen. nov*

Indicimonas (Latin description in relation to the indigo blue color of the marine oligotrophic areas)

Members of the genus *Ca.* Indicimonas are mesophilic, photoheterotrophic marine bacteria, with a scavenging way of life, with the capability of using urea, 2-AEP, fucose, polyamines and creatine. Performing glycolysis and using the pyruvate carboxylase for the anaplerotic CO2 fixation. Based on ANI values (90%), 3 different species have been identified. Genomes belonging to this genus inhabitants the central SPG: GCA_902515745.1; GCA_902518895.1; GCA_902522865.1; GCA_902524385; GCA_902537325.1; GCA_902541215.1; GCA_902571865.1; GCA_902573325.1; GCA_902574475.1; GCA_902582755.1; GCA_902584805.1; GCA_902588745.1; GCA_902595805.1; GCA_902602885.1; GCA_902612825.1; GCA_902614085.1; GCA_902614375.1; GCA_902615745.1; GCA_902620285.1; GCA_902620425.1; GCA_902621165.1; GCA_902621305.1; GCA_902623255.1; GCA_902521315.1; GCA_902540265.1; GCA_902575375.1; GCA_902577905.1; GCA_902600225.1; GCA_902614395.1; GCA_902615625.1 ; GCA_902618015.1; GCA_902585605.1; GCA_902621725.1; GCA_003215235.1; GCA_902554185.1; GCA_902628835.1; GCA_902621855.1; GCA_902524385.1; GCA_003282155.1; and GCA_902525035.1. The GC content for the different species ranges from 27% to 30%. Cells are rod shaped with an average cell size of 0.6 ± 0.1 µm (n=38) (Fig. 1 A inset). This genus was detected in the central SPG in densities of 1.4x10^4^ to 2.6x10^4^ cells ml^-1^.

*Description of Ca. Indicimonas spp.*

*Candidatus* Indicimonas nautili (Latin description of Nautilus name of Captain Nemo’s submarine)

This species was detected in open water samples from the central ultra-oligotrophic SPG, at the top 20 m preferentially, in December 2015 and January 2016. With an estimated GC content between 28% and 29.5%. Genomes belonging to this species are: GCA_003215235.1; GCA_902554185.1

*Candidatus* Indicimonas poseidonii (latin description in honor to Poseidon, Greek god of Seas)

This species was detected in open water samples from the central ultra-oligotrophic SPG, at the top 20 m preferentially, in December 2015 and January 2016. With an estimated GC content of 28%. Genome belonging to this species: GCA_902621855.1.

*Candidatus* Indicimonas neptunia (latin description in honor to Neptune, Roman god of Seas)

This species was detected in open water samples from the central ultra-oligotrophic SPG, at the top 20 m preferentially, in December 2015 and January 2016. The estimated genome size is around 1.6 Mb with a GC content around 29%. Genomes belonging to this species are: GCA_902515745.1; GCA_902518895.1; GCA_902522865.1; GCA_902524385; GCA_902537325.1; GCA_902541215.1; GCA_902571865.1; GCA_902573325.1; GCA_902574475.1; GCA_902582755.1; GCA_902584805.1; GCA_902588745.1; GCA_902595805.1; GCA_902602885.1; GCA_902612825.1; GCA_902614085.1; GCA_902614375.1; GCA_902615745.1; GCA_902620285.1; GCA_902620425.1; GCA_902621165.1; GCA_902621305.1; GCA_902623255.1; GCA_902521315.1; GCA_902540265.1; GCA_902575375.1; GCA_902577905.1; GCA_902600225.1; GCA_902614395.1; GCA_902615625.1 ; GCA_902618015.1; GCA_902585605.1; GCA_902621725.1; GCA_902628835.1;; GCA_902524385.1; GCA_003282155.1; and GCA_902525035.1.

**Supplementary figures**

Figure S1. A) Map of the sampling stations of the SO245 across the SPG. Pale yellow dots indicate stations used in this study. B) Contour plot of the total cell counts (TCC) in cells per milliliter, enumerated by microscopy from depths 0-200 m. Black vertical lines indicate the stations of interest and black diamonds correspond to depths investigated. C) Photosynthetically active radiation (PAR), contour lines represent the TCC. D) UV radiation at the central SPG, contour lines represent the TCC

Figure S2. A) Taxonomic classification of the MAGs obtained from the central SPG at the phylum level. B) The NMDS plot highlights the significant influence of depth on the distribution of the SPG community. The analysis shows that the community composition of the nine Illumina metagenomes is more similar when considered in relation to their respective depths.

Figure S3. Extended data of the relative abundance of the genomes detected in the SPG. A) Represents the AEGEAN169 SPG population. B) SAR11 detected genomes in the SPG. C) SAR116. D) SAR86. The pronounced partitioning of the different microorganisms within the water column is clearly visible in this image.

Figure S4. Consensus phylogenetic tree of the AEGEAN169 clade based on 16S rRNA gene sequences from the PacBio metagenomes. Dark blue branches indicate members of the AEGEAN169 clade that reside in the SPG.

Figure S5. A) Read recruitment plot of metagenomic reads from station 8 at 20 m depth mapped to *Ca.* Nemonibacter GCA_902527045, pale pink area on the right panel indicates the count of base pair with at least 90% similarity. B) Read recruitment plot of metagenomic reads from station 8 at 20 m depth mapped to *Ca.* Indicimonas GCA_003282155, pale pink area on the right panel indicates the count of base pair with at least 90% similarity. C) Plot of AAI vs. percentage of shared proteins in *Ca*. Nemonibacter. D) Plot of AAI vs. percentage of shared proteins in *Ca*. Indicimonas. Both plots indicate that different coexisting members share a high percentage of the genome, thus complicating the efforts of assembly and recovery of AEGEAN169 MAGs. E) AAI matrix plot of the most abundant AEGEAN169-clade II SAGs in the central SPG. The values were always above 80% within each group and below 50% between them, suggesting that *Ca*. Nemonibacter and *Ca*. Indicimonas clades could be considered as two different genera. F) Pairwise AAI calculated between AEGEAN169 and SAR11 genomes had average values around 41% to 42%, below the order boundary, suggesting that AEGEAN169 and SAR11 belong to different orders.

Figure S6. Representation of the detected housekeeping genes transcripts normalized using the biological scaling normalization method for the different clades, stations and depths. The values were used to calculate the fold-change of the expression of the different features in the different clades. Most of the transcripts belonging to *Ca*. Nemonibacter and *Ca*. Indicimonas clades were retrieved from the first 20 m.

Figure S7. Alignment of the protein sequences of the PRs detected in MAGs and SAGs abundant in the SPG and known references. The conserved aspartic acid (D) at position 97 relative to the sequence positions of Gamma-proteobacterium Hot 75m4 (first red box) suggests the proton-pumping activity of the PR. The presence of a conserved glutamine (Q) or leucine (L) at position 105 (second red box) suggests a blue or green-absorbing PR, respectively. The color of the amino acid background from white to dark blue indicates how conserved they are in the alignment.

Figure S8. Phylogenetic tree of PRs detected in *Ca*. Nemonibacter, *Ca*. Indicimonas, SAR11, SAR116, SAR86 most abundant genomes in the SPG, and known references. Red boxes indicate the taxonomic affiliation of the PR.

Figure S9. Representation of the three different pathways responsible for anaplerotic CO2 fixation observed in the different SPG clades studied showing the integration of phosphoenolpyruvate and pyruvate in the TCA cycle (black). The pyruvate carboxylase pathway (in blue) was exclusive to the *Ca*. Nemonibacter, *Ca*. Indicimonas. The phosphoenolpyruvate (PEP) carboxylase pathway (in green) was identified in *Prochlorococcus*, the majority of SAR11 metagenome-assembled genomes (MAGs), and a limited number of SAR86 representatives. The PEP carboxykinase pathway (in red) was annotated in SAR116, the majority of SAR86, and a few SAR11 representatives.

Figure S10. Matrix plot generated to visualize the results of the search for the different transport system using HMM models detected in the different clades studied, showing the number of distinct features per predicted protein.

Figure S11. A) Non-phosphorylating fucose degradation pathway detected in *Ca*. Indicimonas. B) Scheme of the most common genomic context for fucosidase genes in *Ca*. Indicimonas. In one of the operons, the aldose/aldo-keto reductase, mandelate racemase, amidohydrolase, and mutarotase were identified, in most cases, preceded by ABC transporters. In the second operon, the remaining three enzymes of the pathway were found (enoyl reductase, fumarylacetoacetate hydrolase, and L-lactate dehydrogenase).

Figure S12. Phylogeny and genomic context for GH29 exo-fucosidases detected in *Ca*. Indicimonas.

Figure S13. Plots representing the percentage of different transporter systems in relation to the total number of predicted proteins that were detected for the key SPG clades. A) ABC transporters. B) TRAP transporters. C) Membrane transport proteins.

**Supplementary tables**

Table S1. Information about the samples collected for this work regarding the station, date and time, latitude and longitude, and volume of seawater filtered.

Table S2. Information about metagenomes and metatranscriptomes, assemblies and bins retrieved in this work.

Table S3. Description of the genomes used to construct the RAxML genomic tree.

Table S4. Enumeration of the HMM models with HMMER, used in this work.

Table S5. Abundance in the prokaryotic fraction of the different genomes belonging to the AEGEAN169, SAR11, SAR116, SAR86, and *Prochlorococcus* clades at the central SPG, calculated as the quotient of the genome’s TAD80 value and the genome equivalent for each metagenome.

Table S6. Core metabolic functions of *Ca*. Nemonibacter, *Ca*. Indicimonas.

Table S7. AEGEAN169 information about genomes present in the SPG and features found in their genomes.

Table S8. SAR11 information about genomes present in the SPG and features found in their genomes.

Table S9. SAR116 information about genomes present in the SPG and features found in their genomes.

Table S10. SAR86 information about genomes present in the SPG and features found in their genomes.

Table S11. *Prochlorococcus* information about genomes present in the SPG and features found in their genomes.

**Supplementary references**

1. Mueller J, Morel A, Frouin R *et al.* *Ocean optics protocols for satellite ocean color sensor validation, revision 4, volume iii: Radiometric measurements and data analysis protocols*. Goddard Space Flight Space Centre, 2003.

2. Organelli E, Claustre H, Bricaud A *et al.* A novel near-real-time quality-control procedure for radiometric profiles measured by Bio-Argo floats: Protocols and performances. *J Atmos Oceanic Technol*. 2016;**33**:937-51 https://doi.org/https://doi.org/10.1175/JTECH-D-15-0193.1

3. Francis TB, Krüger K, Fuchs BM *et al. Candidatus* Prosiliicoccus vernus, a spring phytoplankton bloom associated member of the *Flavobacteriaceae*. *Sys Appl Microbiol*. 2019;**42**:41-53 https://doi.org/https://doi.org/10.1016/j.syapm.2018.08.007

4. Rodriguez-R L, Konstantinidis K. Nonpareil: A redundancy-based approach to assess the level of coverage in metagenomic datasets. *Bioinformatics*. 2014;**30**:629-35 https://doi.org/10.1093/bioinformatics/btt584

5. Ondov BD, Treangen TJ, Melsted P *et al.* Mash: Fast genome and metagenome distance estimation using MinHash. *Genome Biol*. 2016;**17**:132 https://doi.org/10.1186/s13059-016-0997-x

6. Gerhardt K, Ruiz-Perez CA, Rodriguez-R LM *et al.* RecruitPlotEasy: An advanced read recruitment plot tool for assessing metagenomic population abundance and genetic diversity. *Front Bioinf*. 2022;**1** https://doi.org/10.3389/fbinf.2021.826701

7. Camacho C, Coulouris G, Avagyan V *et al.* Blast+: Architecture and applications. *BMC Bioinformatics*. 2009;**10**:421 https://doi.org/10.1186/1471-2105-10-421

8. El-Gebali S, Mistry J, Bateman A *et al.* The Pfam protein families database in 2019. *Nucleic Acids Res*. 2019;**47**:D427-D32 https://doi.org/10.1093/nar/gky995

9. Jones P, Binns D, Chang H-Y *et al.* InterProScan 5: Genome-scale protein function classification. *Bioinformatics*. 2014;**30**:1236-40 https://doi.org/10.1093/bioinformatics/btu031

10. Schwengers O, Jelonek L, Dieckmann MA *et al.* Bakta: Rapid and standardized annotation of bacterial genomes via alignment-free sequence identification. *Microb Genomics*. 2021;**7** https://doi.org/https://doi.org/10.1099/mgen.0.000685

11. Seemann T. Prokka: Rapid prokaryotic genome annotation. *Bioinformatics*. 2014;**30**:2068-69 https://doi.org/10.1093/bioinformatics/btu153

12. Aramaki T, Blanc-Mathieu R, Endo H *et al.* KofamKOALA: KEGG Ortholog assignment based on profile HMM and adaptive score threshold. *Bioinformatics*. 2020;**36**:2251-52 https://doi.org/10.1093/bioinformatics/btz859

13. Kanehisa M, Sato Y, Morishima K. BlastKOALA and GhostKOALA: KEGG tools for functional characterization of genome and metagenome sequences. *J Mol Biol*. 2016;**428**:726-31 https://doi.org/https://doi.org/10.1016/j.jmb.2015.11.006

14. Stamatakis A. RAxML version 8: A tool for phylogenetic analysis and post-analysis of large phylogenies. *Bioinformatics*. 2014;**30**:1312-13 https://doi.org/10.1093/bioinformatics/btu033

15. Delmont TO, Quince C, Shaiber A *et al.* Nitrogen-fixing populations of Planctomycetes and Proteobacteria are abundant in surface ocean metagenomes. *Nat Microbiol*. 2018;**3**:804-13 https://doi.org/10.1038/s41564-018-0176-9

16. Thiele S, Fuchs BM, Amann RI Identification of microorganisms using the ribosomal RNA approach and fluorescence *in situ* hybridization. In: Frimmel FaW, P (ed.). *Treatise on water science*, Amsterdam, The Netherlandes: Oxford Academic Press, Elsevier. 171-89. Retreived from https://www.sciencedirect.com/science/article/abs/pii/B9780444531995000567

17. Reintjes G, Tegetmeyer HE, Burgisser M *et al.* On-site analysis of bacterial communities of the ultraoligotrophic South Pacific Gyre. *Appl Environ Microbiol*. 2019;**85**:e00184-19 https://doi.org/10.1128/AEM.00184-19

18. Bennke CM, Reintjes G, Schattenhofer M *et al.* Modification of a high-throughput automatic microbial cell enumeration system for shipboard analyses. *Appl Environ Microbiol*. 2016;**82**:3289-96 https://doi.org/10.1128/AEM.03931-15

19. Ducret A, Quardokus EM, Brun YV. MicrobeJ, a tool for high throughput bacterial cell detection and quantitative analysis. *Nat Microbiol*. 2016;**1**:16077 https://doi.org/10.1038/nmicrobiol.2016.77

20. Schindelin J, Arganda-Carreras I, Frise E *et al.* Fiji: An open-source platform for biological-image analysis. *Nat Methods*. 2012;**9**:676-82 https://doi.org/10.1038/nmeth.2019

21. Brüwer JD, Orellana Luis H, Sidhu C *et al.* *In situ* cell division and mortality rates of SAR11, SAR86, *Bacteroidetes*, and *Aurantivirga* during phytoplankton blooms reveal differences in population controls. *mSystems*. 2023;**8**:e01287-22 https://doi.org/10.1128/msystems.01287-22
