# Supplementary figures and images for: Niche differentiation within bacterial key-taxa in stratified surface waters of the Southern Pacific Gyre
Source: ISME J. 2024 Aug 3;18(1):wrae155. doi: 10.1093/ismejo/wrae155 (PMC11366302; doi:10.1093/ismejo/wrae155)

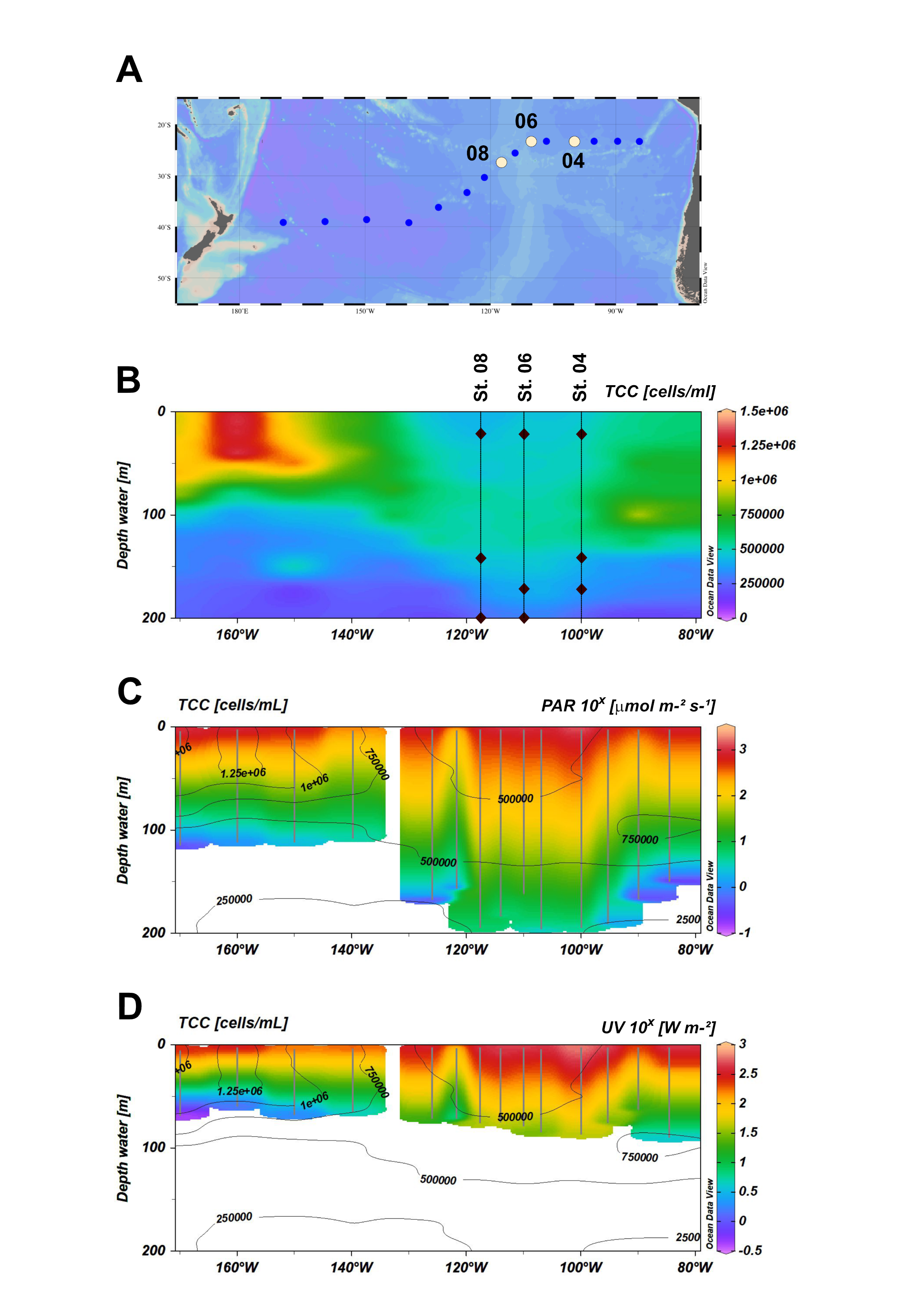

Supplement: suppl_wrae155 [file suppl_wrae155.zip › Fig S1.tiff]

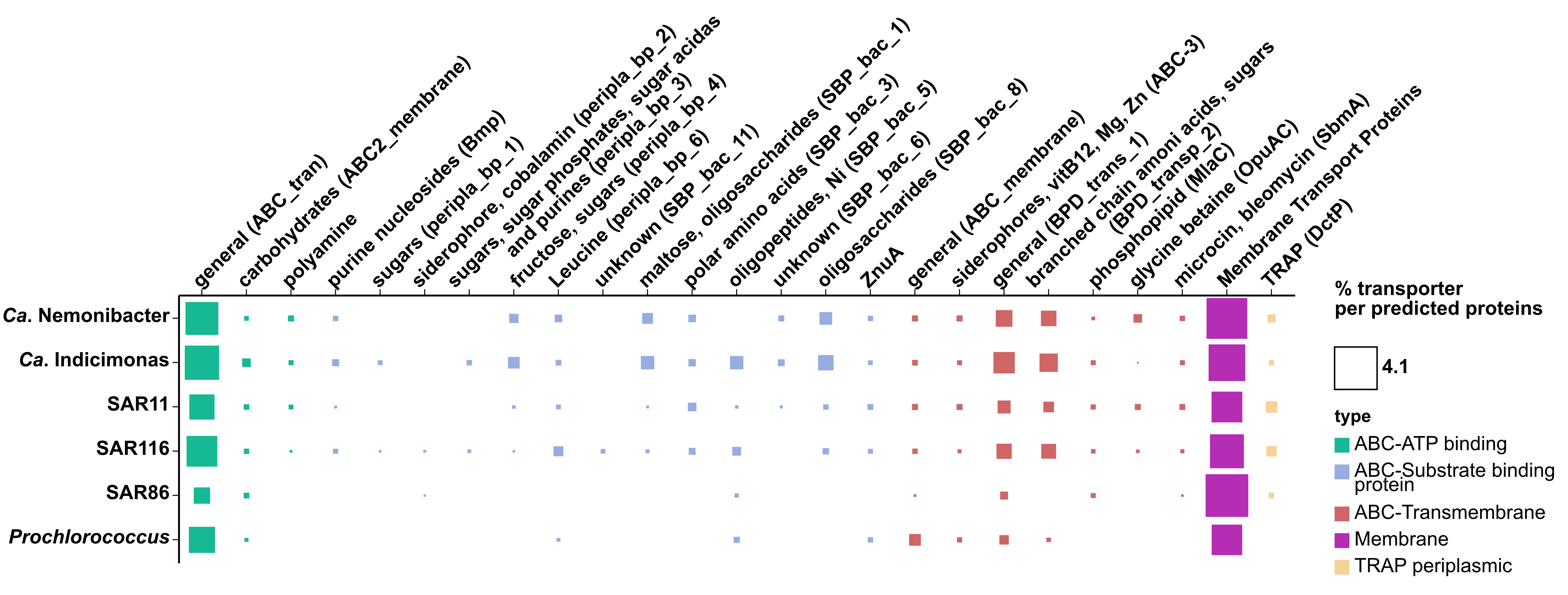

Supplement: suppl_wrae155 [file suppl_wrae155.zip › Fig S10.tiff]

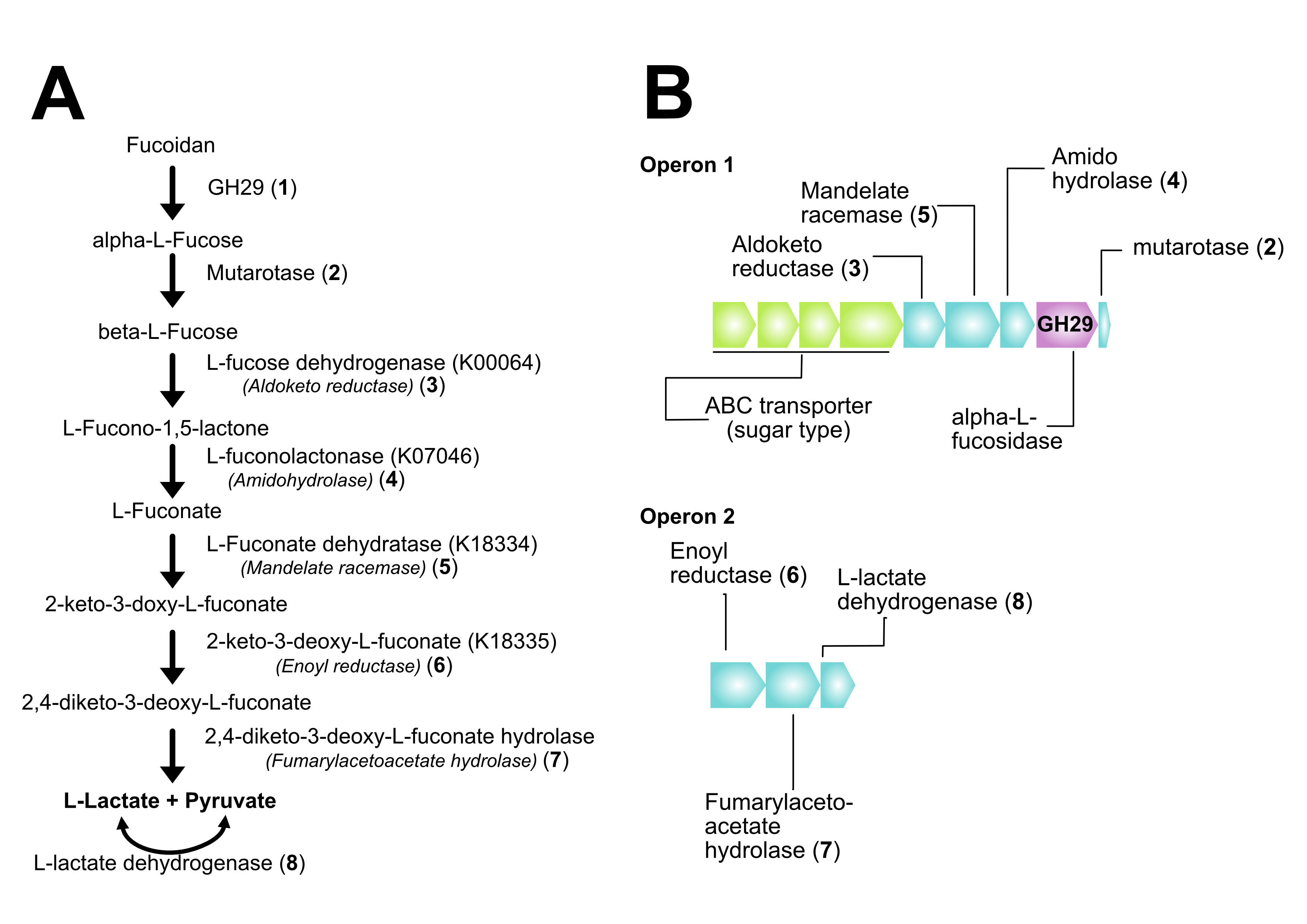

Supplement: suppl_wrae155 [file suppl_wrae155.zip › Fig S11.tiff]

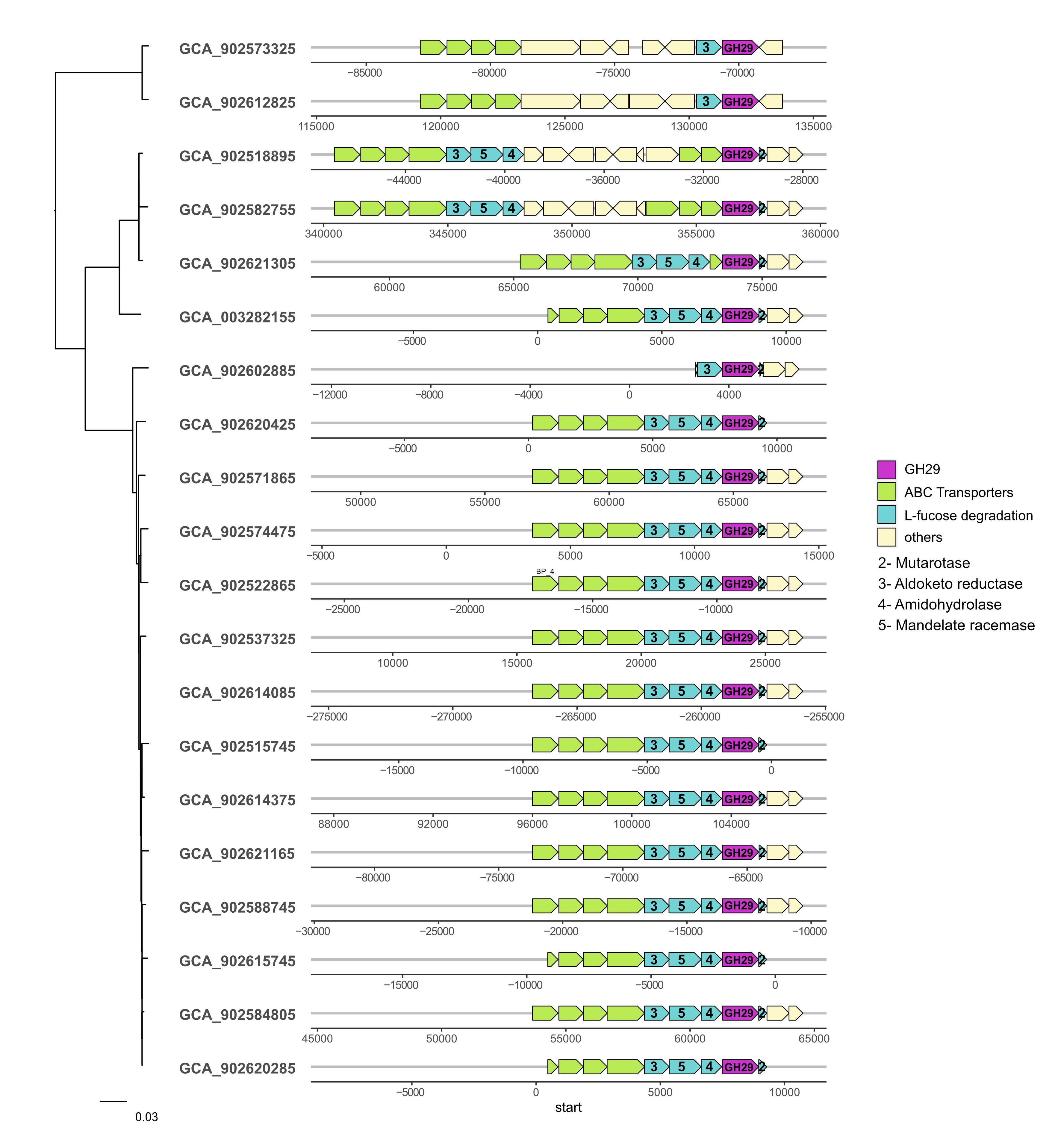

Supplement: suppl_wrae155 [file suppl_wrae155.zip › Fig S12.tiff]

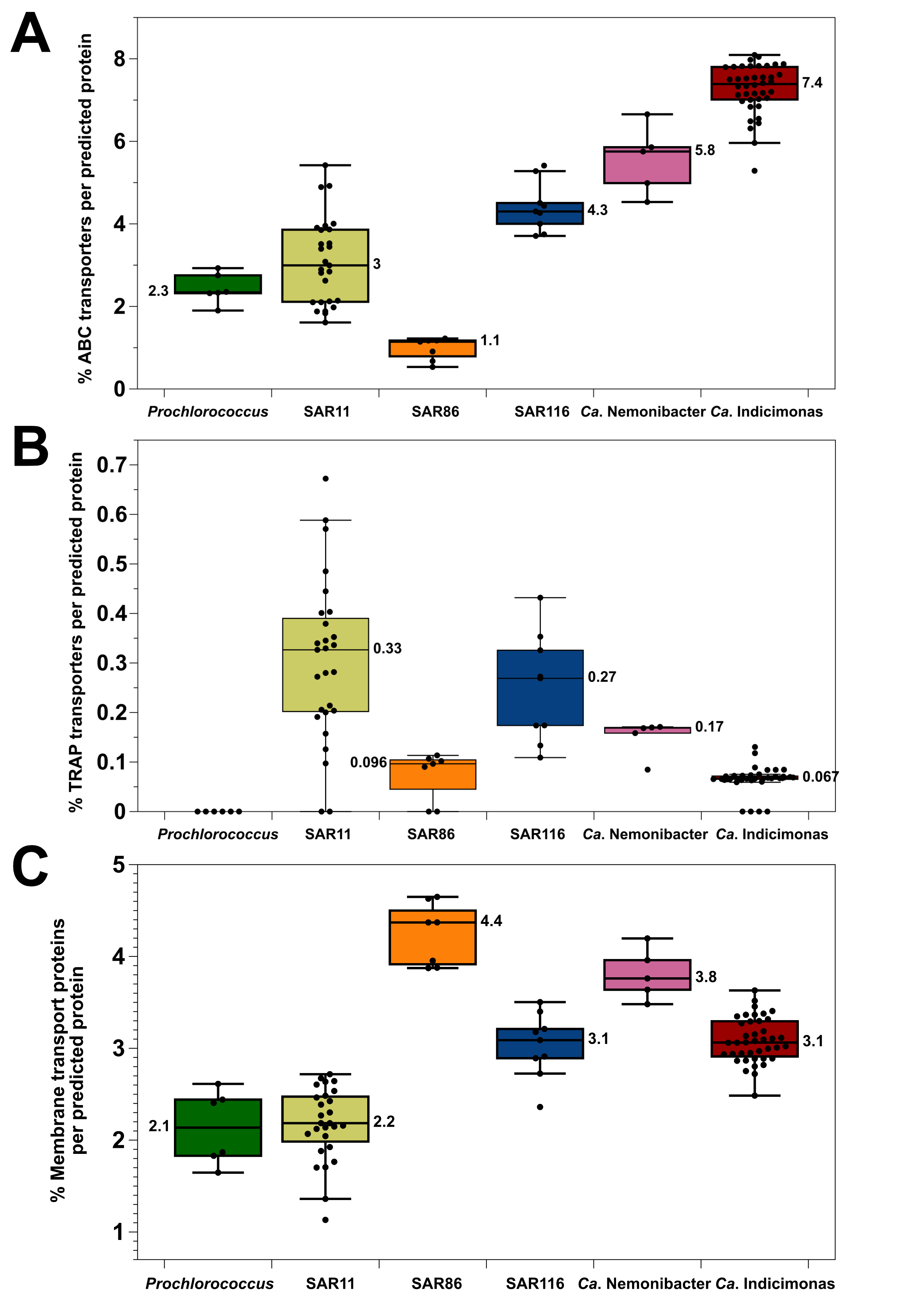

Supplement: suppl_wrae155 [file suppl_wrae155.zip › Fig S13.tiff]

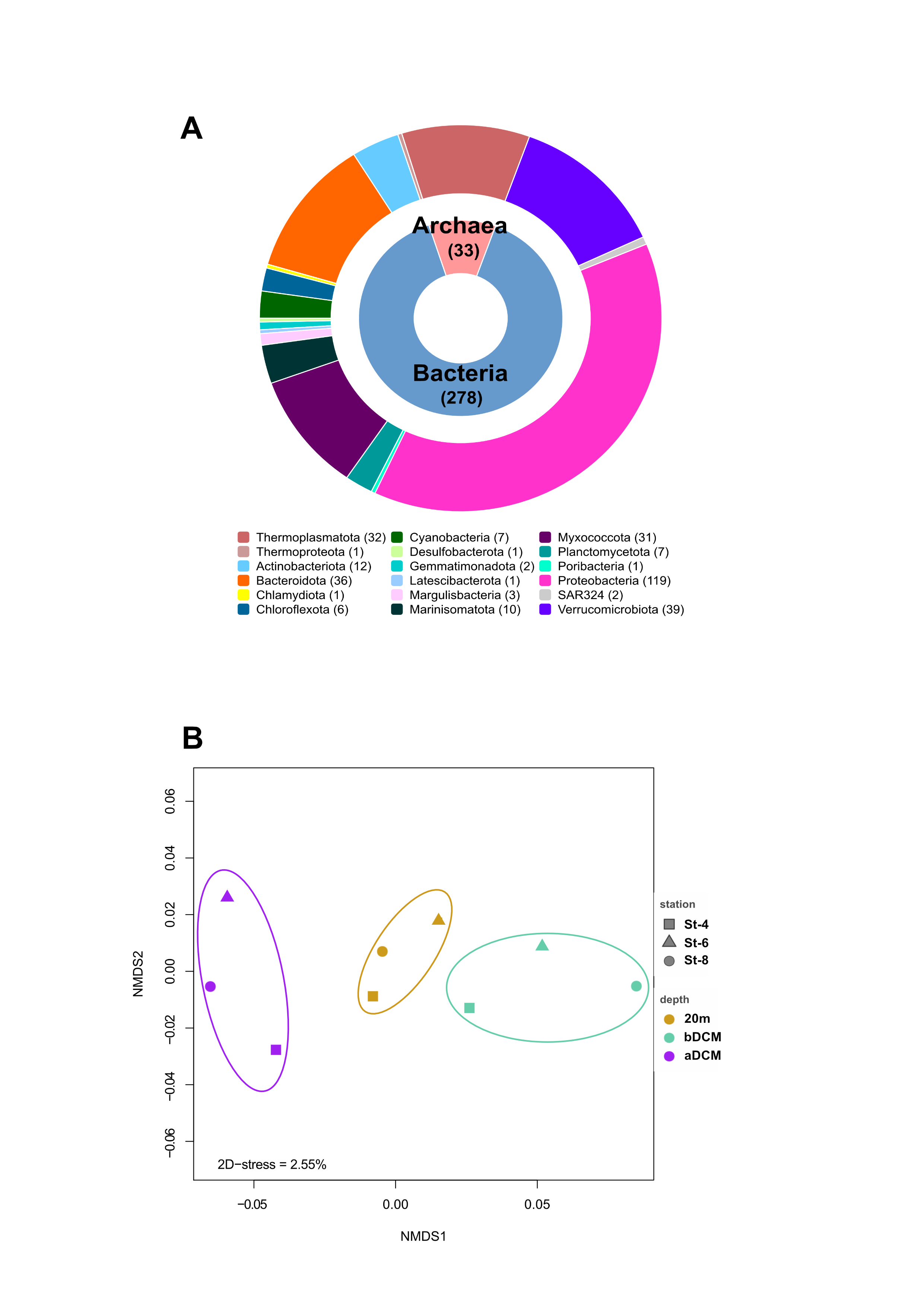

Supplement: suppl_wrae155 [file suppl_wrae155.zip › Fig S2.tiff]

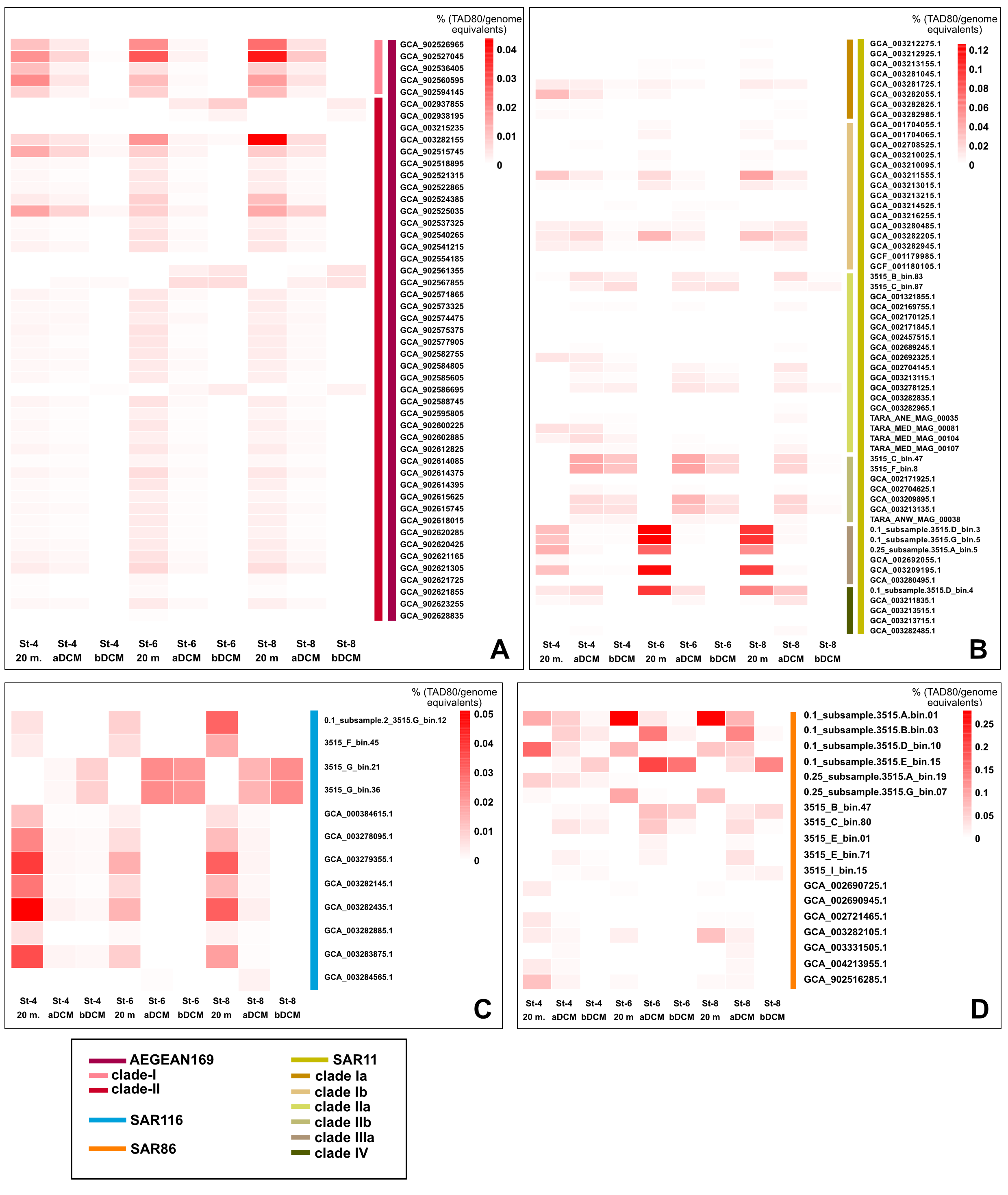

Supplement: suppl_wrae155 [file suppl_wrae155.zip › Fig S3.tiff]

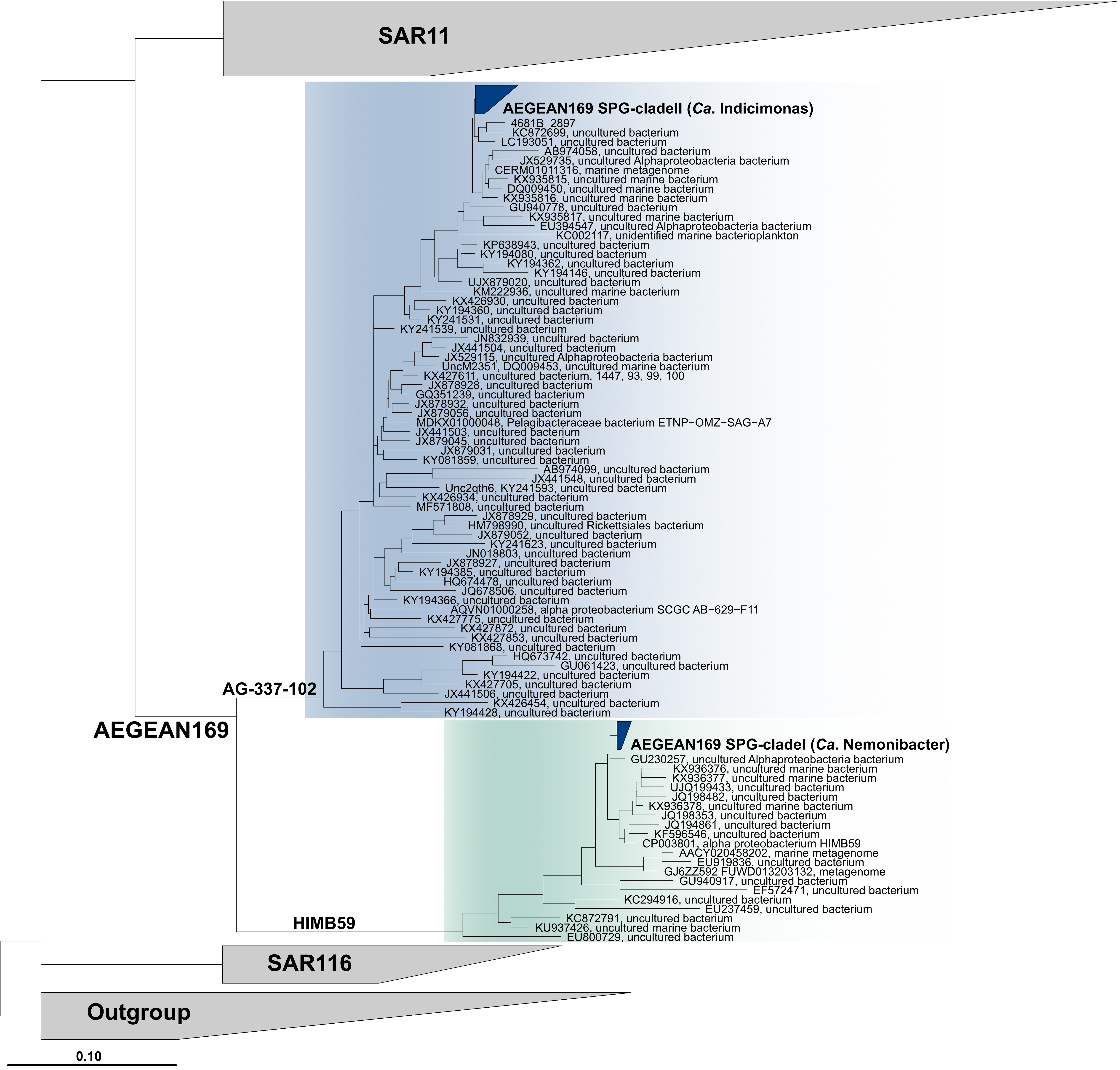

Supplement: suppl_wrae155 [file suppl_wrae155.zip › Fig S4.tiff]

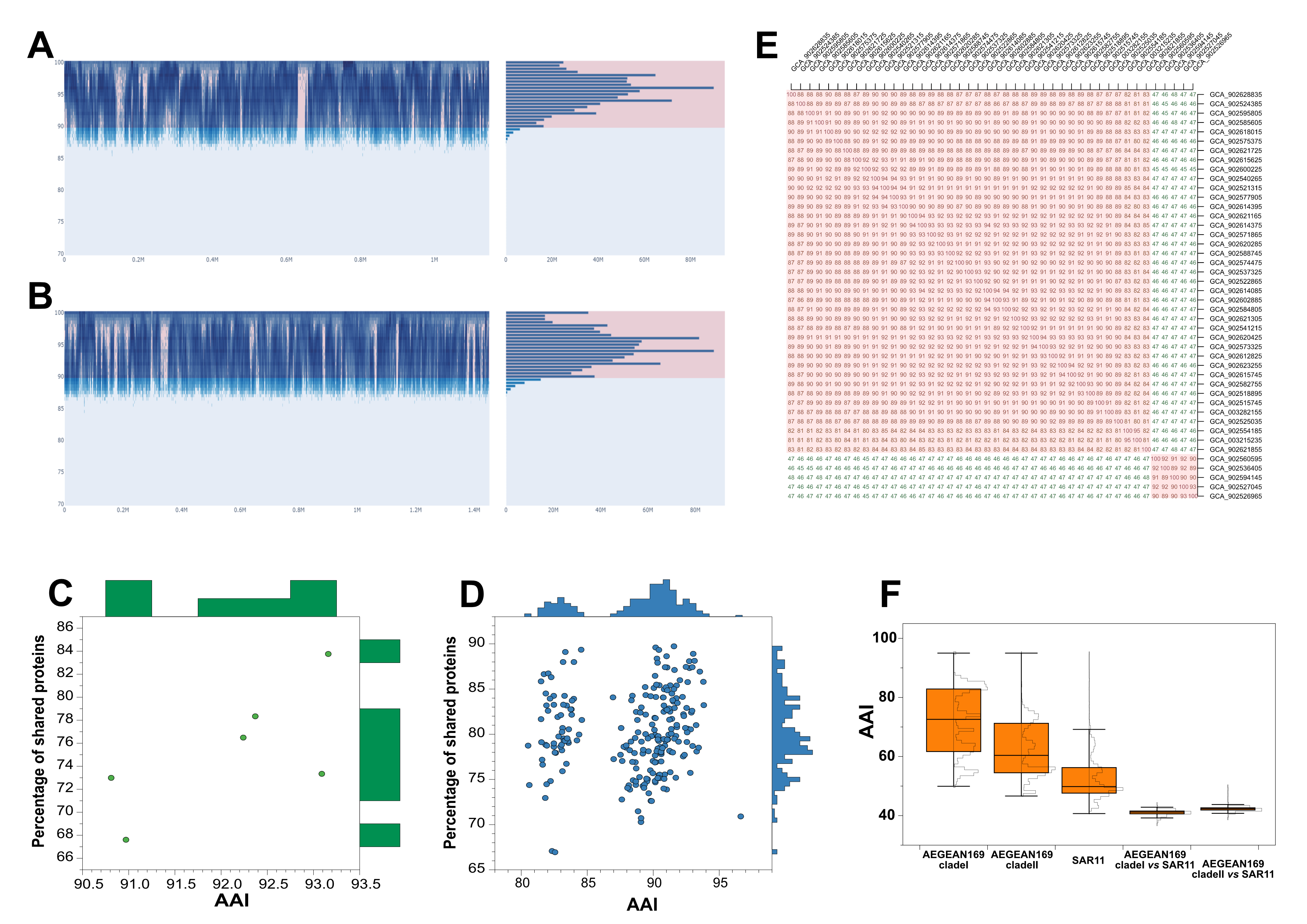

Supplement: suppl_wrae155 [file suppl_wrae155.zip › Fig S5.tiff]

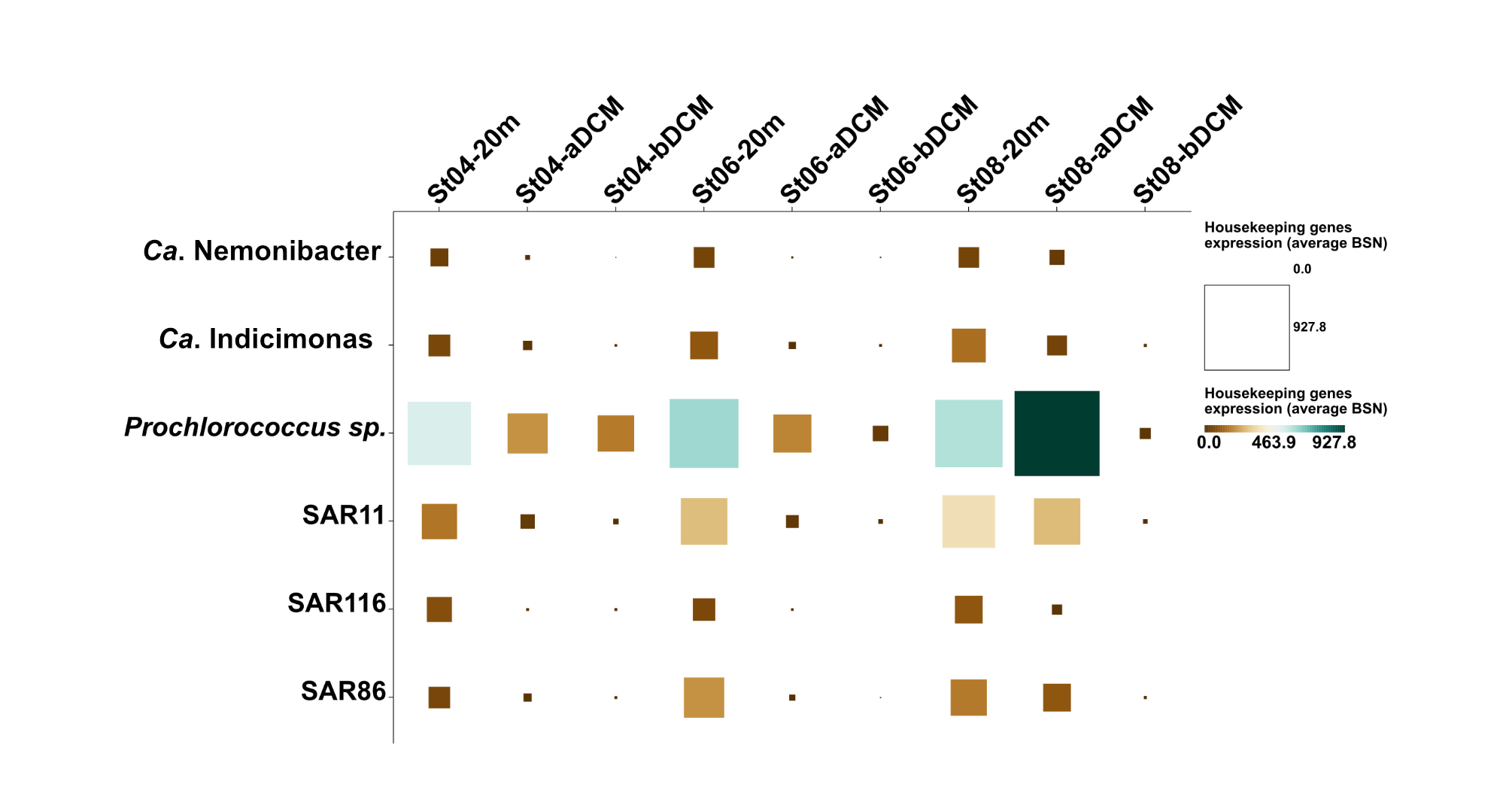

Supplement: suppl_wrae155 [file suppl_wrae155.zip › Fig S6.tiff]

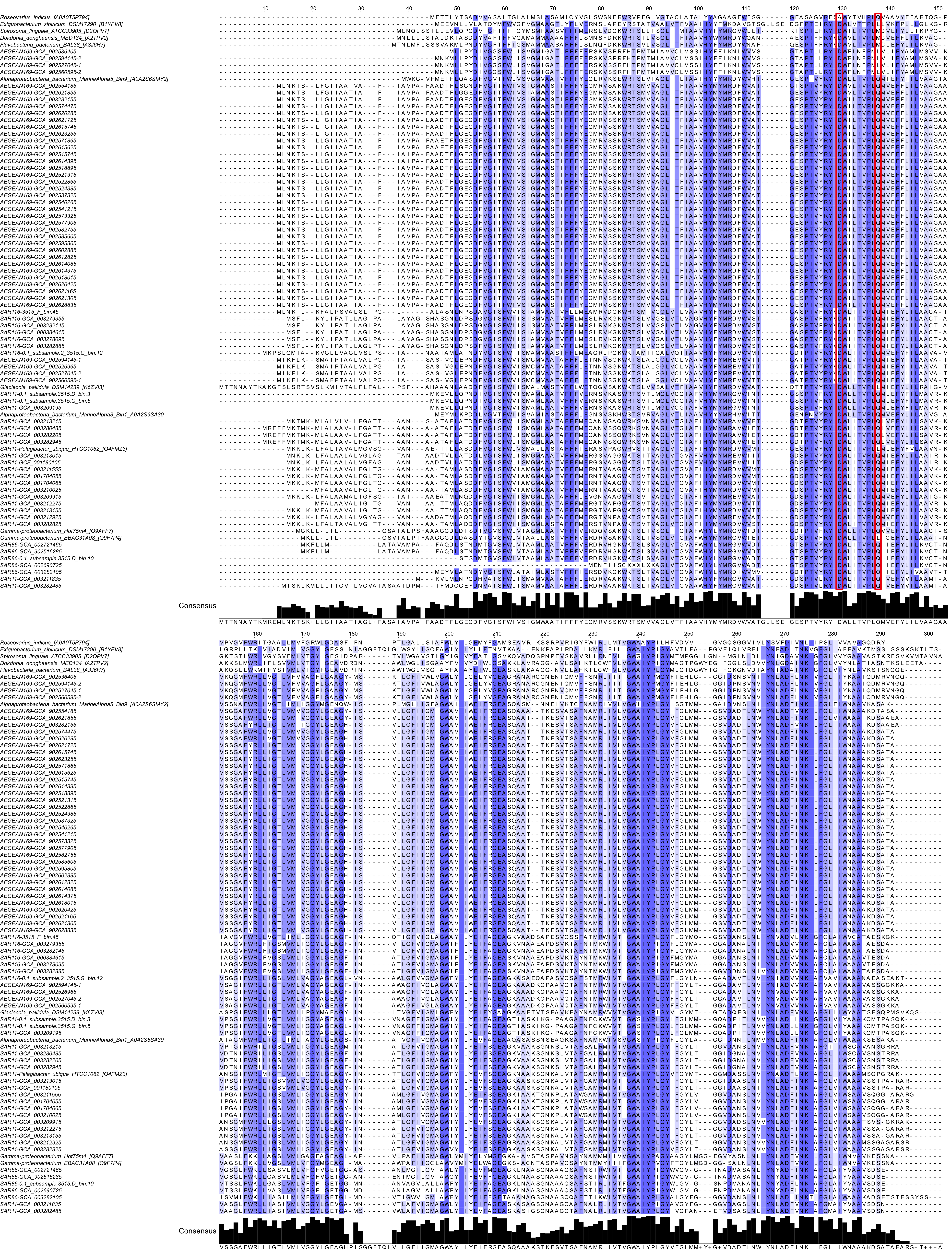

Supplement: suppl_wrae155 [file suppl_wrae155.zip › Fig S7.tiff]

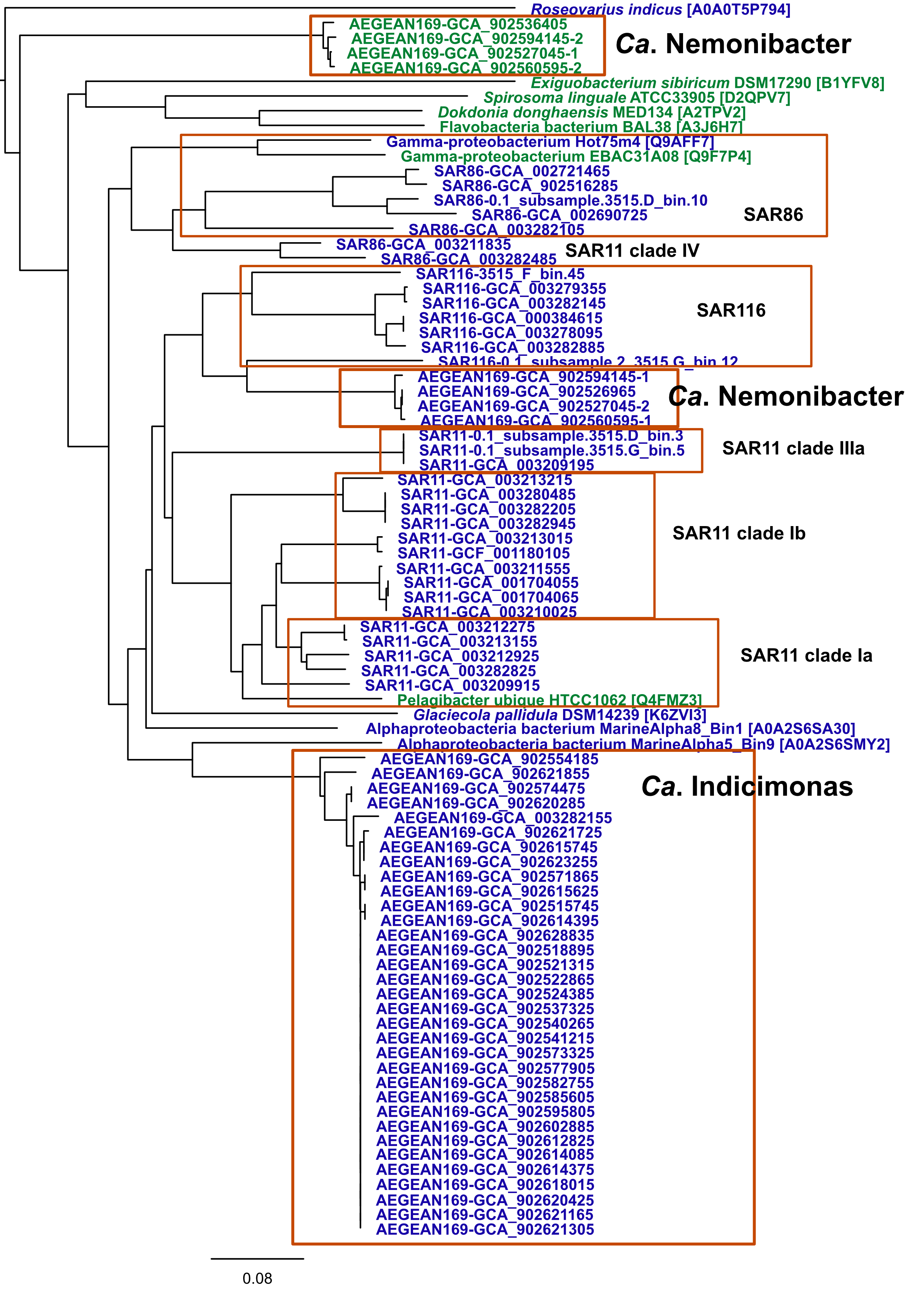

Supplement: suppl_wrae155 [file suppl_wrae155.zip › Fig S8.tiff]

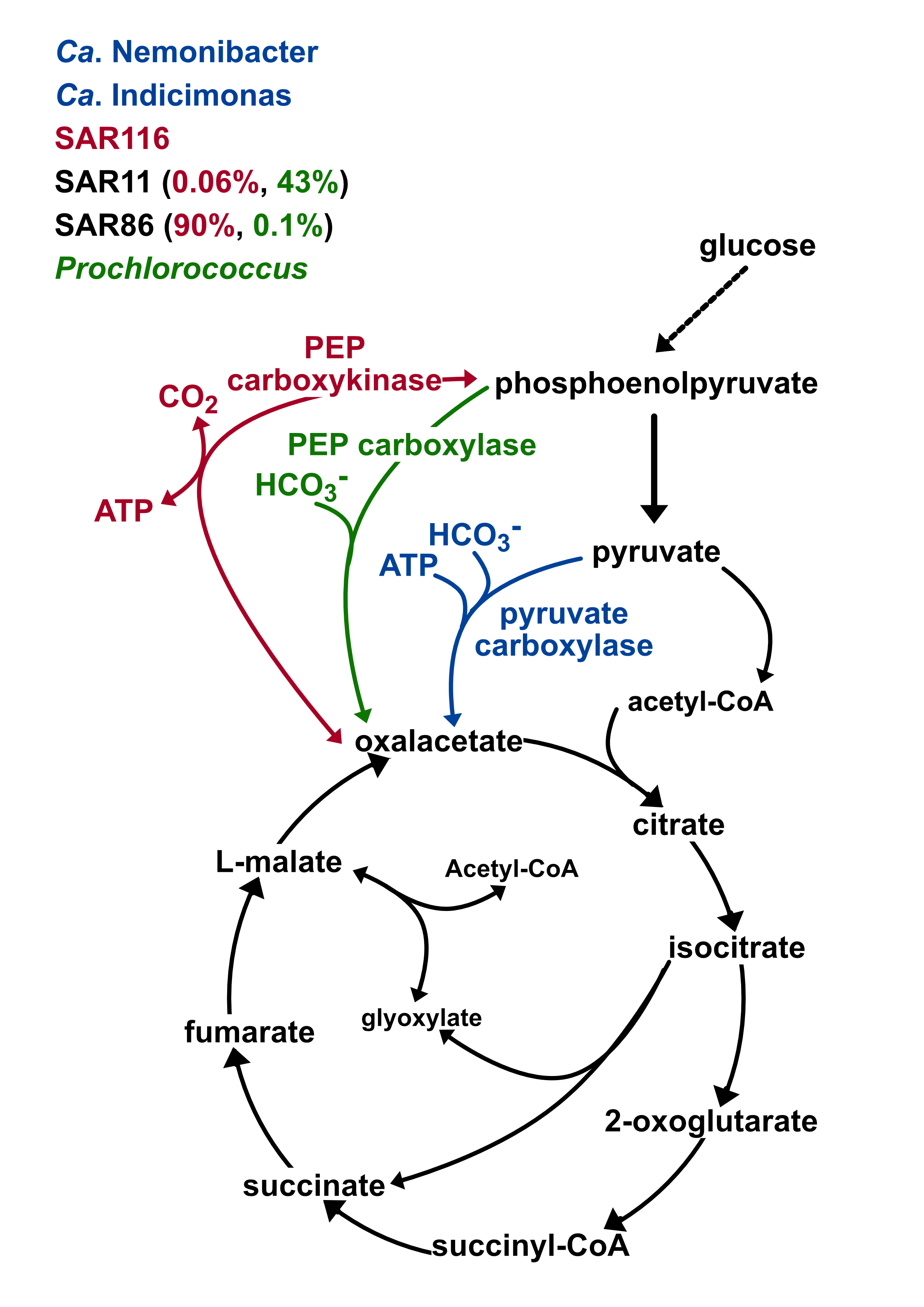

Supplement: suppl_wrae155 [file suppl_wrae155.zip › Fig S9.tiff]
